# Rna Buffering Fluorogenic Probe for Nucleolar Morphology Stable Imaging And Nucleolar Stress‐Generating Agents Screening

**DOI:** 10.1002/advs.202309743

**Published:** 2024-02-07

**Authors:** Wenchao Jiang, Qinglong Qiao, Jie Chen, Pengjun Bao, Yi Tao, Yinchan Zhang, Zhaochao Xu

**Affiliations:** ^1^ CAS Key Laboratory of Separation Science for Analytical Chemistry Dalian Institute of Chemical Physics Chinese Academy of Sciences 457 Zhongshan Road Dalian 116023 China; ^2^ University of Chinese Academy of Sciences Beijing 100049 China

**Keywords:** buffering, fluorogenic probes, nucleolus morphology, RNA. imaging

## Abstract

In the realm of cell research, membraneless organelles have become a subject of increasing interest. However, their ever‐changing and amorphous morphological characteristics have long presented a formidable challenge when it comes to studying their structure and function. In this paper, a fluorescent probe **Nu‐AN** is reported, which exhibits the remarkable capability to selectively bind to and visualize the nucleolus morphology, the largest membraneless organelle within the nucleus. **Nu‐AN** demonstrates a significant enhancement in fluorescence upon its selective binding to nucleolar RNA, due to the inhibited twisted intramolecular charge–transfer (TICT) and reduced hydrogen bonding with water. What sets **Nu‐AN** apart is its neutral charge and weak interaction with nucleolus RNA, enabling it to label the nucleolus selectively and reversibly. This not only reduces interference but also permits the replacement of photobleached probes with fresh ones outside the nucleolus, thereby preserving imaging photostability. By closely monitoring morphology‐specific changes in the nucleolus with this buffering fluorogenic probe, screenings for agents are conducted that induce nucleolar stress within living cells.

## Graphical abstract

A buffering nucleolar RNA fluorogenic probe **Nu‐AN** is developed. This innovative probe exhibits selective fluorescence enhancement by inhibiting TICT and reducing hydrogen bond effect upon binding to RNA. Owning to its neutral charge and weak interaction with nucleolar RNA, **Nu‐AN** selectively reversibly labels the nucleolus, tailored for nucleolar morphology imaging, and facilitating the screening of nucleolar stress‐generating agents.

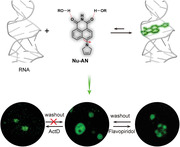

## 1 Introduction

The nucleolus, the largest membraneless organelle within the cell nucleus, has gained increasing recognition for its diverse physiological and pathological functions, which are inherently linked to its morphology.^[^ ^1^ ^]^ Primarily serving as the hub for ribosome biogenesis, the nucleolus also possesses non‐ribosomal functions, including the regulation of mitosis, cell cycle control, and participation in cellular responses to stress.^[^ ^2^ ^]^ The morphological aspects of the nucleolus, encompassing its shape, size, and the number of nucleoli within a cell nucleus, reflect its physiological activity, and correlate with various human diseases, such as cancer, neurodegenerative conditions, and aging.^[^ ^3^ ^]^ For instance, in cancer cells, the heightened need for ribosome biogenesis, and protein synthesis due to rapid cell growth and proliferation leads to larger, more numerous, and irregularly shaped nucleoli compared to those in normal cells. Additionally, nucleolus size exhibits a strong association with lifespan, with smaller nucleoli being a hallmark of cellular longevity.^[^ ^4^ ^]^ Moreover, changes in nucleolar morphology become particularly pronounced under conditions of nucleolar stress, providing valuable insights for disease diagnosis, and drug screening.^[^ ^5^ ^]^ Consequently, the visualization of nucleolar morphology serves as a valuable tool to explore the functions of the nucleolus and unravel the connections between alterations in nucleolar morphology and various diseases. For example, Lundberg et al. systematically dissected the human nucleolar proteome using confocal microscopy and identified a potential fourth nucleolar subcompartment, the nucleolus rim.^[^ ^6^ ^]^ Similarly, using high‐resolution live‐cell microscopy, Chen et al. identified a distinctive nucleolar sub‐region, and the periphery of the dense fibrillar component (PDFC).^[^ ^7^ ^]^

Nonetheless, fluorescence imaging of nucleolar morphology encounters challenges, primarily due to the dynamic, and amorphous structure of this membraneless organelle.^[^ ^8^ ^]^ The nucleolus comprises three distinct components: fibrillar centers (FC) housing ribosomal RNA genes (rDNA) for ribosomal RNA (rRNA) transcription, dense fibrillar components (DFC) containing actively processed, and modified rRNA transcripts surrounding the FC, and granular components (GC) at the nucleolar outermost region, which hold fully assembled ribosomal subunits ready for export to the cytoplasm.^[^ ^1c^ ^]^ The most straightforward strategy for nucleolus imaging is to tag nucleolar proteins with genetically encoded fluorescent proteins, a method offering precision but burdened by limitations like low transfection efficiency, protein overexpression, and complex procedures. Alternatively, due to the widespread presence of RNA in the nucleolus, imaging often relies on fluorescent staining of RNA using external small molecule dyes. However, many of these small molecule probes possess positively charged or ionizable alkaline groups, leading to strong electrostatic interactions with RNA.^[^ ^9^ ^]^ These groups can cause undesired background staining in the cytoplasm,^[^ ^10^ ^]^ staining other organelles, and affecting cell function. Therefore, there is a pressing need for fluorescent probes for nucleolar morphology imaging that can selectively identify the overall nucleolus structure without interfering with nucleolar function and exhibit high photostability for dynamic nucleolus imaging.

**Figure 1:**
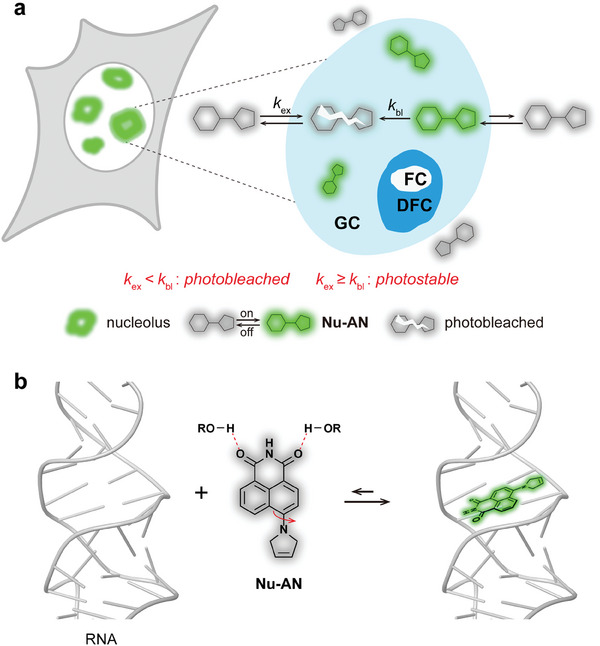
a) **Nu‐AN** reversibly labels the nucleolus minimizing interference with the nucleolus and forming a buffer pool of dye outside the nucleolus enabling stable imaging. b) Mechanism of fluorescence activation upon **Nu‐AN** binding to RNA.

In our work, we introduced a novel fluorogenic probe **Nu‐AN** for visualizing nucleolar morphology (**Figure** **1**). **Nu‐AN** stands out due to its neutral charge and the absence of protonatable basic groups, resulting in weak interactions with RNA, and enabling reversible RNA binding. When applied to live cells, **Nu‐AN** effectively and reversibly labels the nucleolus, while avoiding undesired accumulation in mitochondria or lysosomes. This reversibility not only minimizes interference with the nucleolus but also creates a reservoir of dye outside the nucleolus (Figure 1a). If probes within the nucleolus become photobleached, probes from the reservoir can be quickly recruited, and ensuring consistent imaging stability. Incorporating a 3‐pyrroline ring into the naphthalimide fluorophore maintains planarity and preserves the characteristic properties of twisted intramolecular charge–transfer (TICT). **Nu‐AN** also exhibits hydrogen bond quenching, a trait seen in traditional naphthalimide dyes.^[^ ^11^ ^]^ Upon binding to RNA, **Nu‐AN** resides within the hydrophobic cavity of RNA, and reducing hydrogen bond effect, ultimately restoring fluorescence (Figure 1b). Consequently, the reversible binding and fluorogenic property of **Nu‐AN** to RNA ensure nucleolar specificity and stable imaging, making it particularly suitable for visualizing nucleolar substructure granule components (GC) without disrupting nucleolar function. Complete nucleolus imaging can be achieved by co‐staining **Nu‐AN** with fluorescent proteins. By observing nucleolar morphology‐specific changes, we could explore the potential for screening nucleolar stress‐inducing agents.

## 2 Results and Discussion

### 2.1 Fluorogenicity for RNA by Suppressing TICT and Reducing Hydrogen Bond Effect

**Figure 2:**
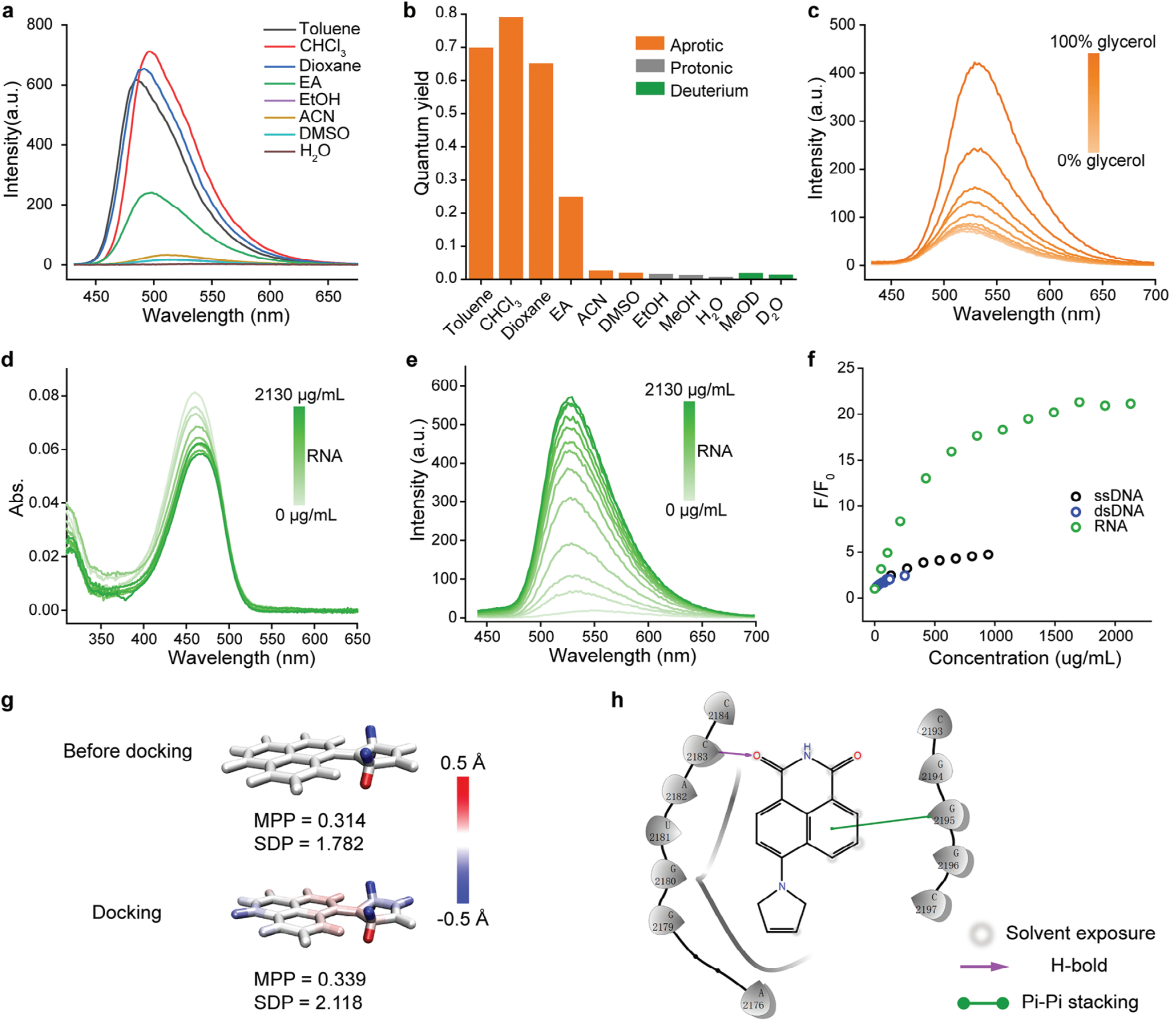
a) Fluorescence spectra of **Nu‐AN** in different solvents. b) Fluorescence quantum yield of **Nu‐AN** in different solvents. c) Fluorescence spectra of **Nu‐AN** in mixed solutions of methanol and glycerol with different volume fractions. d) Absorption spectra of **Nu‐AN** in the presence of different concentrations of RNA. e) Fluorescence spectra of **Nu‐AN** in the presence of different concentrations of RNA. f) Corresponding fluorescent intensity enhancement as a function of nucleic acid (RNA, ssDNA, and dsDNA) concentration. g) Molecular configuration before and after docking with RNA. Coloring diagram of MPP and atomic SDP of **Nu‐AN**. h) 2D diagram of interactions between **Nu‐AN** and RNA molecular docking.

**Nu‐AN** was synthesized according to Scheme S1 (Supporting Information), and its absorption and fluorescence spectra were examined in various solvents (**Figure** **2**a; Figure S1a and Table S1, Supporting Information). The absorption and emission wavelengths of **Nu‐AN** experienced a redshift with increasing solvent polarity, attributable to its intramolecular charge–transfer properties. This shift was accompanied by a reduction in fluorescence intensity. The analysis of quantum yields of **Nu‐AN** in aprotic, pro tonic, and deuterium solvents indicated fluorescence quenching in highly polar and protic solvents (Figure 2b; Table S1, Supporting Information). Conversely, **Nu‐AN** exhibited a high quantum yield in low‐polarity solvents, also showing a slightly enhanced quantum yield in deuterated solvents compared to their protic analogues (0.020 in CD_3_OD vs 0.014 in CH_3_OH; 0.015 in D_2_O vs 0.007 in H_2_O). These findings suggest that intramolecular charge–transfer may be the primary factor in fluorescence quenching, while hydrogen bonding between **Nu─AN** and the solvent also played a role in quenching the fluorescence to some extent. Twisted intramolecular charge–transfer (TICT), the major nonradiative de‐excitation pathways, and is typically readily formed in naphthalimide dyes.^[^ ^11d^ ^]^ Accordingly, we measured the fluorescence intensity in mixtures of different ratios of methanol and glycerol. As the proportion of glycerol increases, the viscosity of the mixed solvent increases, which suppresses TICT formation, and resulting in enhanced fluorescence (Figure 2c; Figure S1b, Supporting Information). Interestingly, in low viscosity mixtures, the quantum yield did not increase significantly, and or even decreased (Table S2 and Figure S2, Supporting Information). The net change in quantum yield hinged on the interplay between TICT inhibition and hydrogen bond quenching. The addition of glycerol inhibited TICT while strengthening hydrogen bond interactions. When the glycerol ratio surpassed 30%, the inhibition of TICT dominated the increase in quantum yield. These findings suggest that the fluorescence of **Nu‐AN** was influenced by both TICT and hydrogen bonds. Enhancing fluorescence was achievable by inhibiting TICT and diminishing the interaction of hydrogen bonds.

Armed with this environmentally sensitive probe, we endeavored to explore the fluorescence response of **Nu‐AN** to nucleic acids. The absorption and emission spectra of **Nu‐AN** in the presence of varying concentrations of yeast RNA were measured. With an increase in RNA concentration, the absorption spectra exhibited a redshift (from 460 to 468 nm) accompanied by a decrease in intensity. Simultaneously, the fluorescence spectra displayed a blueshift (from 550 to 529 nm) and a gradual increase in fluorescence intensity (Figure 2d,e; Table S3, Supporting Information). This shift in spectra was attributed to the inhibition of TICT in **Nu‐AN** within hydrophobic microenvironments. To demonstrate the selectivity of the probe for RNA, fluorescent titration of **Nu‐AN** with dsDNA, and ssDNA were performed. **Nu‐AN** exhibited a 4.7 fold and 2.4 fold fluorescence enhancement in response to dsDNA and ssDNA, respectively, compared to a significant 22 fold increase with RNA (Figure 2f), demonstrating **Nu‐AN** was remarkable selective for RNA over DNA in solutions.

To clarify the changes in the absorption and emission spectrum upon **Nu‐AN** binding to RNA, as well as to understand the mechanism of the interaction with RNA, quantum chemical calculations with Gaussian 16 software, and molecular docking simulation calculation with Schrödinger software were conducted. Two molecular metric parameters, namely the molecular planarity parameter (MPP) and the span of deviation from plane (SDP), have been established as effective indicators for characterizing the planarity of molecules.^[^ ^12^ ^]^ It was observed that before docking, both MPP (0.314 vs 0.339) and SDP (1.782 vs 2.118) of **Nu‐AN** were smaller compared to post‐docking results (Figure 2g). Possibly due to the reduction in planarity of **Nu‐AN** upon binding to RNA, the conjugation degree of **Nu‐AN** decreased, and leading to a reduction in absorption intensity in the presence of RNA. This suggests that the RNA binding pocket constrained the configuration of **Nu‐AN** to a certain extent, consequently inhibiting TICT, and subsequently enhancing fluorescence. Moreover, molecular docking analysis revealed that **Nu‐AN** was situated within the hydrophobic core of the RNA, with only a small portion exposed to the surrounding solvent environment (Figure 2h). In more detail, a *π*–*π* stacking interaction was observed between the naphthalene ring of **Nu‐AN** and probe formed a hydrogen bond with another nucleotide residue. Despite the recognized potential of hydrogen bonding to quench the fluorescence of **Nu‐AN**, the hydrogen bond formed within the RNA hydrophobic pockets had a less impact compared to being in a complete solvent. Consequently, suppressing TICT and reducing hydrogen bonds ensured fluorogenic properties of **Nu‐AN** upon binding to RNA.

### 2.2 Fluorogenic Probe for the Nucleolus

**Figure 3:**
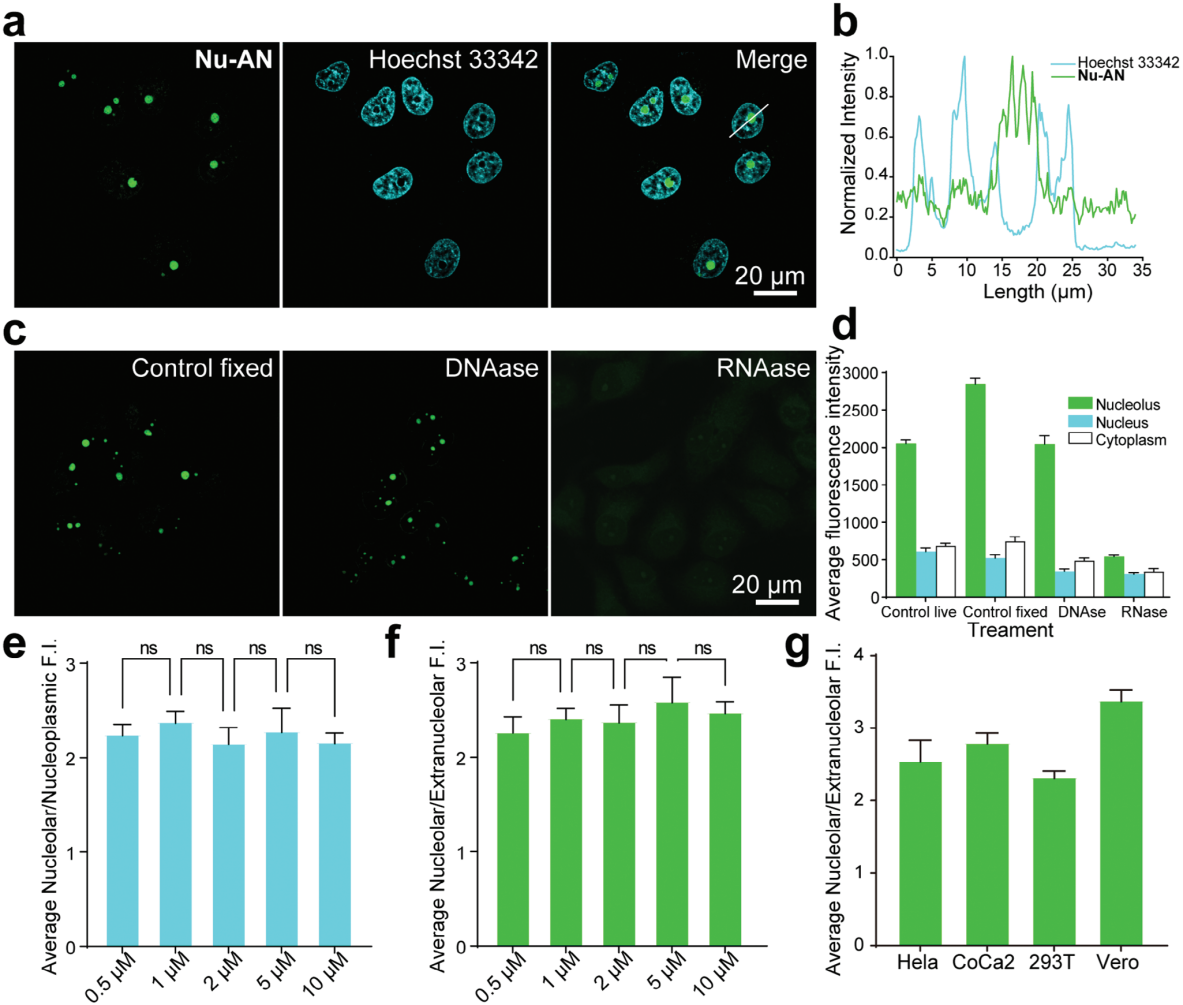
a) Wash‐free imaging of living Hela cells. Hela cell was incubated with 3 µm Hoechst 33342 for an hour, followed by 2 µm **Nu‐AN** for 15 min. b) Normalized fluorescence intensity distribution in the region of interest. c) Fluorescent imaging of fixed Hela cells untreated, treated with DNase, or RNase. d) Average fluorescence intensity of living Hela cells, fixed Hela cells, fixed Hela cells treated with DNase, or RNase. e) The ratio of the average fluorescence intensity of the nucleolus to the nucleoplasm at different concentrations in living Hela cells. f) The ratio of the average fluorescence intensity of the nucleolus to the extranucleolar cellular region at different concentrations in living Hela cells. g) The ratio of the average fluorescence intensity of the nucleolus to the extranucleolar cellular region in different cell lines.

Encouraged by fluorogenic properties of **Nu‐AN** for RNA, and considering that the nucleolus is rich in ribosomal RNA that constitute over 80% of the total cellular RNA,^[^ ^13^ ^]^ we proceeded to explore its potential for wash‐free imaging of the nucleolus in living cells. Hela cell was incubated with the Hoechst 33342 nuclear dye for an hour, followed by **Nu‐AN** for 15 min before confocal imaging without the washing step. Distinct green fluorescence from **Nu‐AN** only appeared in areas devoid of blue fluorescence stained with Hoechst 33342 inside the nuclei (**Figure** **3**a,b). The co‐staining experiment not only demonstrated its ability to perform dual color imaging with Hoechst 33342 but also confirmed its localization within the nucleolus. Typically, dyes that stain nucleoli are prone to displaying an unavoidable mitochondrial or lysosomal fluorescence background in the cytoplasm.^[^ ^10a–c^ ^]^ However, to our delight, **Nu‐AN** did not exhibit mitochondrial or lysosomal fluorescence signals, as evidenced by co‐localization experiments with commercial dyes (Figure S4, Supporting Information), which was attributed to the fact that **Nu‐AN** had no net charge or alkaline groups. To further verify its selectivity for staining the nucleolus, enzyme digestion experiments using ribonuclease (RNase), and deoxyribonuclease (DNase) were conducted following the method reported in previous literature.^[^ ^10b^ ^]^ As shown in figure 3c, the nucleoli exhibited substantial fluorescence in untreated fixed cells and DNase‐digested cells. However, upon treatment with RNase, the fluorescent signal completely disappeared. Moreover, the ratio of the average fluorescence intensity of the nucleolus to the nucleoplasm in fixed cells and cells treated with DNase was as high as 5 times (Figure 3d), whereas the ratio of commercial RNA dyes, SYTO RNASelect Green (SYTO), was only 2.3 times.^[^ ^14^ ^]^ All of these findings strongly indicated that **Nu‐AN** demonstrates high selectivity toward nucleoli in living cells.

Subsequently, the cytotoxicity of **Nu‐AN** was investigated by MTT assays. The results revealed that over 90% of Hela cells survived after 24 h (20 µm **Nu‐AN** incubation), demonstrating the low toxicity of **Nu‐AN** toward cultured cell lines (Figure S5, Supporting Information). These findings collectively supported the potential use of **Nu‐AN** for wash‐free imaging of living cell nucleoli.

To further demonstrate that **Nu‐AN** could serve as a fluorogenic probe for nucleoli, we measured the concentration dependence of nucleolar imaging under confocal fluorescence microscopy. The probe could image nucleoli well and exhibited a considerable signal‐to‐noise ratio even at high concentrations (ranging from 0.5 to10 µm, Figure S6, Supporting Information). The ratios of the average fluorescence intensity of the nucleolus to the nucleoplasm and the average fluorescence intensity of the nucleolus to the extranucleolar cellular region showed no significant difference at different concentrations, and both signal‐to‐noise ratios reached 2.5 times (Figure 3e,f). To verify the generality of the probe for imaging nucleoli in living cells, two cancer cell lines, Hela cell, and CoCa2 cell, and two normal cell lines, 293T cell and Vero cell, were selected for parallel staining experiments. The results showed that all four cell lines displayed excellent nucleolus staining selectivity (Figure S7, Supporting Information), with the ratio of the average fluorescence intensity of the nucleolus to the extranucleolar cellular region of >2.5 times. Particularly in Vero cells, this ratio was >3 times (Figure 3g). These robust results strongly indicated that **Nu‐AN** was a general fluorogenic probe for the nucleolus, holding potential for exploring nucleolar morphological changes.

### 2.3 Reversible Binding Enables Photostable Imaging without Interference

An excellent nucleolar probe not only necessitates highly selective staining of the nucleolus but also demands that the probe does not interfere with the normal physiological activities of the nucleolus. Additionally, for prolonged imaging, the dye must demonstrate photostability during the imaging process. The reversible binding of dyes to the target is a crucial feature that minimizes interference with the target and ensures imaging photostability.^[^ ^15^ ^]^ We anticipated, due to its molecularly neutral chemical nature, that **Nu‐AN** could achieve reversible labeling of the nucleolus by binding reversibly to nucleolar RNA. When the probes within the nucleolus were photobleached, they could be rapidly replaced by intact probes outside the nucleolus, and ensuring consistent imaging stability.^[^ ^15^, ^16^ ^]^ To verify the reversible binding of nucleoli by **Nu‐AN**, Hela cells were incubated with **Nu‐AN** for wash‐free imaging, and nucleoli were clearly observed. Upon washing out the dyes with fresh culture medium, the fluorescence of nucleoli disappeared and reappeared with re‐introduction of the dyes (Figure S8, Supporting Information). Given its reversible labeling of the nucleolus, we investigated the photostability of the dye.

**Figure 4:**
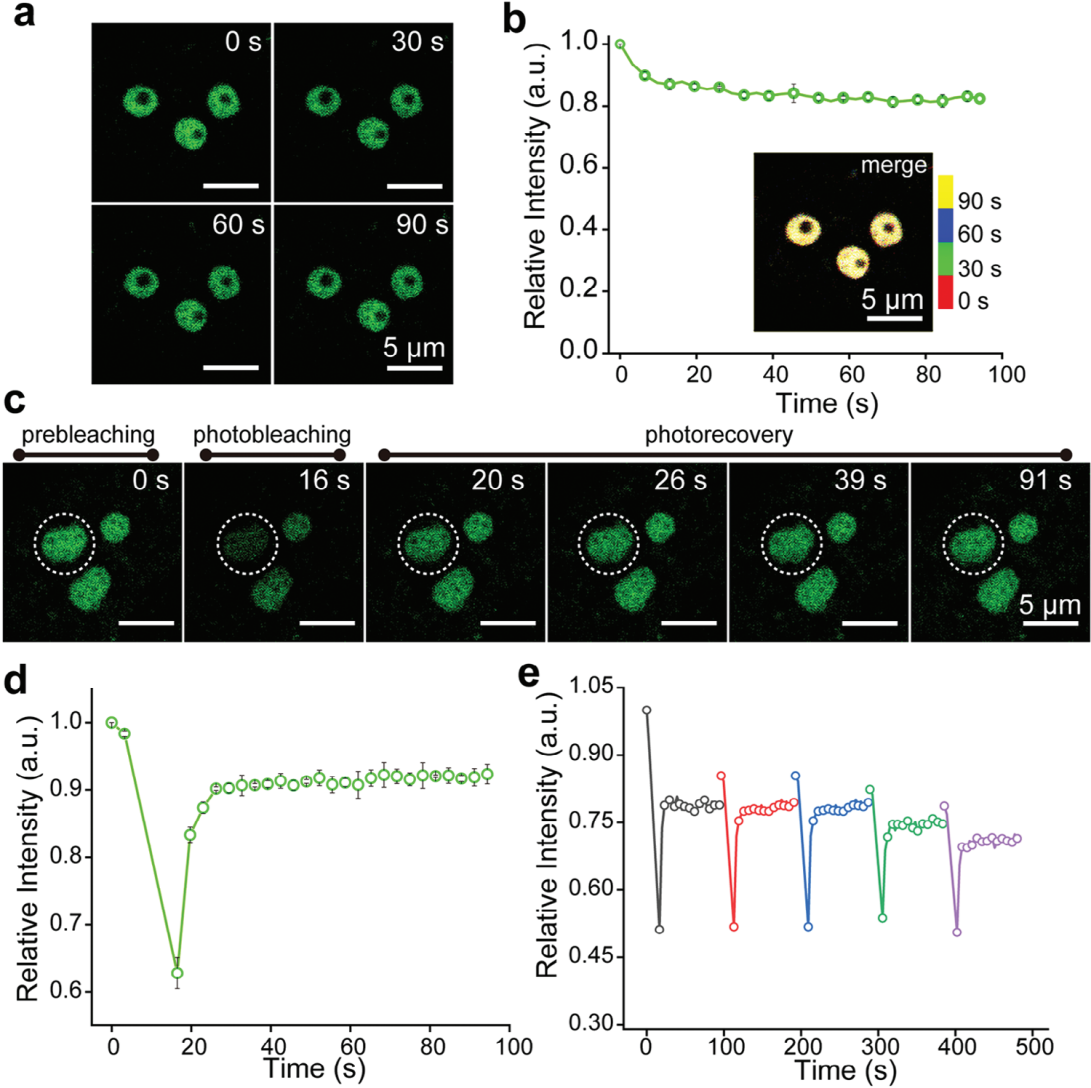
a) Time‐lapse confocal imaging of living Hela cells stained with **Nu‐AN** with no time interval. b) Relative intensity of **Nu‐AN** in the living Hela cell correspond to the imaging process in a). Nucleoli are assigned different pseudo colors at 0 s (red), 30 s (green), 60 s (blue), 90 s (yellow), and the merged image is in the inset. c) Confocal images of living Hela cell stained with **Nu‐AN** during FRAP processes. d) Relative intensity is plotted ver‐sus time (s) in the region of interest labelled in c) during FRAP processes. e) Relative intensity is plotted versus time (s) in the region of interest during five cycles of FRAP processes.

Time‐lapse confocal imaging without any time intervals showed that the fluorescence intensity initially reduced to 80% and then stabilized (**Figure** **4**a,b). In addition, the images of nucleoli at different times (0 s, 30 s, 60 s, 90 s) could completely overlap without any morphological changes. These outcomes demonstrated that **Nu‐AN** maintained stable imaging and did not interfere with nucleolar morphology. We further explored the kinetics of reversible binding of the dye to the nucleolus by fluorescence recovery after photobleaching (FRAP) experiments. Significant FRAP was observed within 10 s (Figure 4c,d). Further, the FRAP experiment was repeated at the same region for five cycles, and the fluorescence intensity of the nucleolus could be restored to 75% (Figure 4f; Figure S9, Supporting Information). It was the rapid exchange of bleached probes on the nucleolus with intact probes in the buffer pool that ensured the stability of nucleolar imaging. Thus, the reversible labeling of **Nu‐AN** to the nucleolus not only did not interfere with nucleolar morphology, but also ensured stable imaging.

### 2.4 Nucleolar Morphology Imaging and Screening of Nucleolar Stress‐Generating Agents

**Figure 5:**
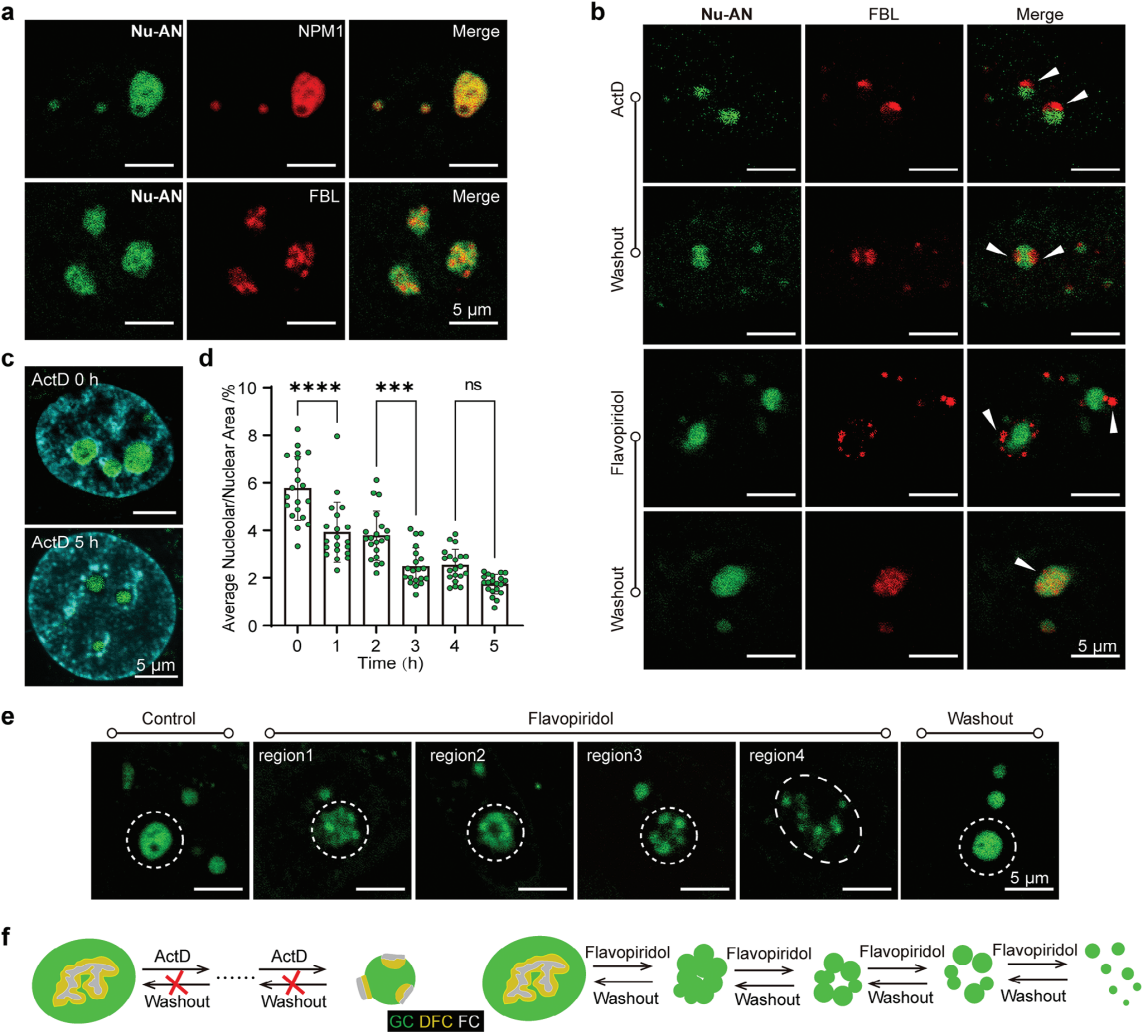
a) Confocal imaging of living Hela cells transiently expressing NPM1‐mCherry or FBL‐mCherry co‐stained with **Nu‐AN**. b) Morphology of nucleoli in living Hela cells treated with anti‐cancer drugs and after drugs washout under confocal microscopy. FBL‐mCherry transiently expressing living Hela cells treated with 8 nM ActD for 4 h or 10 µm Flavopiridol for 1 h, and then co‐stained with 2 µm **Nu‐AN** for imaging. The drug was washed off and cultured Hela cell for another 4 h, and then 2 µm **Nu‐AN** was added for imaging. c) Living Hela cells treated with 8 nM ActD at 0 h and 5 h, the co‐stained with 3 µm Hoechst 33342 and 2 µm **Nu‐AN** for confocal imaging. d) The ratio of the average area of nucleoli to nuclei of Hela cells treated with 8 nM ActD for different times and stained with 2 µm **Nu‐AN**. e) Confocal imaging of living Hela cells in different region. Hela cells was treated 10 µm Flavopiridol for 1 h and stained with 2 µm **Nu‐AN**. f) The model of morphological changes of nucleolus treated with ActD (left) or Flavopiridol (right).

The nucleolus, being a membraneless organelle formed through the liquid–liquid phase separation of multiple components, exhibits highly dynamic, and uncertain morphology. This includes variations in its shape, size, and the number of nucleoli within a cell nucleus aligning with its complex function. Notably, nucleolar morphological changes can be readily induced by various environmental factors such as heat shock, chilling stress, UV radiation, oxidants, and or other agents capable of inducing nucleolar stress.^[^ ^5a^ ^]^ As a result, monitoring nucleolar morphological alterations has become a significant method for disease diagnosis and drug screening.^[^ ^17^ ^]^ Based on specific, non‐interference labeling and stable imaging performance of **Nu‐AN** for the nucleolus, we further explored its utility in nucleolar morphology imaging and drug screening. The nucleolus is morphologically considered to consist of FC, DFC, and GC, which could be labelled separately with three proteins: transcription factor UBF for the FC, fibrillarin (FBL) for the DFC, nucleophosmin (NPM1) for the GC.^[^ ^18^ ^]^ First, we verified the selectivity of **Nu‐AN** for nucleolar substructure. By co‐staining NPM1 or FBL fused with mCherry fluorescent protein with **Nu‐AN**, we found that the green fluorescence of **Nu‐AN** completely coincided with the fluorescence of NMP1 and the intensity profiles of the linear regions were closely synchronized. However, the fluorescence signals of **Nu‐AN** and FBL were not consistent, and there was no obvious co‐localization (**Figure** **5**a; Figure S10, Supporting Information). Although both the GC and DFC contain RNA, the probe selectively stained GC, while the fluorescence of the probe in DFC was dim, which may be due to the fact that the GC is rich in rRNA and ribosomal subunits while DFC mainly contain pre‐rRNA and processing factors of early RNA.^[^ ^2^ ^]^ Based on the above experimental facts and considering that FC is a transcription site com‐posed of rDNA and transcription‐related factors, **Nu‐AN** was proved to specifically stain GC. The size of the nucleolus is mainly determined by the GC, which represents the overall shape of the nucleolus.^[^ ^19^ ^]^ To visualizing the global nucleolar morphology, we consequently co‐stained cells with **Nu‐AN** for GC and FBL‐mCherry for DFC (Figure 5b).

Different types of drugs may induce different changes in the morphology of the nucleolus due to their different mechanisms of action. Actinomycin D (ActD) is an RNA synthesis inhibitor that inhibits the transcription of RNA polymerase I by inserting into the G/C base rich region of rDNA, thereby inhibiting RNA synthesis.^[^ ^5a^ ^]^ Flavopiridol is an ATP competitive cyclin dependent kinase (CDKs) that inhibits rRNA synthesis by causing RNA polymerase I to dissociate from rDNA.^[^ ^20^ ^]^ Subsequently, we explored whether **Nu‐AN** could monitor nucleolar morphological changes induced by these two drugs. Two groups of Hela cells co‐staining with NPM1‐mCherry and **Nu‐AN** were treated with ActD for 4 h or Flavopiridol for 1 h, respectively. After drug treatment, the signals of NPM1‐mCherry and **Nu‐AN** of the two groups of cells completely overlapped under the confocal microscope (Figure S11, Supporting Information), indicating that **Nu‐AN** also exhibits excellent labeling capability for the GC under drug treatment conditions.

Drug treatment usually causes damage to the integrity of the nucleolus, potentially causing simultaneous phase separation in different regions of the nucleolus. To comprehensively observe the morphological changes of nucleoli, Hela cells expressing FBL‐mCherry were treated with drugs, and then co‐stained with **Nu‐AN** to labeling DFC and GC simultaneously (Figure 5b; Figure S12, Supporting Information). For cells treated with ActD, the DFC was separated from the GC to form “caps”^[^ ^21^ ^]^ at the nucleolar periphery (Figure 5b). When the drug was washed off and cultured for another 4 h, the nucleolar morphology still could not be restored. In addition, the ratio of the average area of nucleoli to the average area of nuclei was significantly reduced from 6% to 2% after 5 h of treatment (Figure 5c,d; Figures S11 and S13, Supporting Information), and the imaging signal‐to‐noise ratio of the nucleolus stained with **Nu‐AN** was also decreased (Figure S14, Supporting Information).

Surprisingly, a different nucleolar morphology changes was observed in Flavopiridol treated cells. The separation between the DFC and the GC was more thorough, to the extent that they were completely detached from each other. Although Flavopiridol exhibited a more severe impact on nucleolar morphology, this effect was reversible. After washing off the drug, the nucleolar morphology could be completely restored after an additional 4 h of incubation (figure 5b). Furthermore, different states of nucleoli were also observed in different fields of view after Flavopiridol treatment and staining with **Nu‐AN** (Figure 5e; Figure S15, Supporting Information), implying that the nucleolus may undergo a gradual transition from an intact and compact state to a loosely separated state, which is consistent with recent results reported in the literature.^[^ ^20^ ^]^ These imaging results have shown that both drugs induce morphological changes in nucleoli, and these changes may be specific.

As depicted in Figure 5f, ActD stimulation led to the formation of stress “caps” and a reduction in nucleolar size. This alteration was found to be irreversible. Conversely, nucleoli exhibited a gradual transition from a compact and intact state to a severely dispersed state under the influence of Flavopiridol. This stimulation was more intense but notably reversible. The distinct reversible properties observed could be attributed to the differing mechanisms of these two drugs. The binding of ActD to rDNA may be irreversible, whereas Flavopiridol, acting as a competitive kinase inhibitor, and may engage in a reversible binding to its target. In general, **Nu‐AN** proves to be a valuable tool in visualizing nucleolar morphology and monitoring dynamic changes in response to drug stimulation. This capability allows for efficient drug screening by observing and analyzing specific morphological alterations in the nucleolus.

## 3 Conclusion

In summary, we have successfully developed a buffering nucleolar RNA fluorogenic probe **Nu‐AN**, tailored for nucleolar morphology imaging, and facilitating the screening of nucleolar stress‐generating agents. This innovative probe exhibited selective fluorescence enhancement by inhibiting TICT and reducing hydrogen bond effect upon binding to RNA. Benefiting from its neutral charge and weak interaction with nucleolar RNA, **Nu‐AN** selectively reversibly labelled the nucleolus not only to reduce interference but also allow for maintaining imaging stability. Notably, **Nu‐AN** enabled the visualization of comprehensive nucleolar morphology in conjunction with fluorescent proteins labeling the nucleolus within living cells. Using **Nu‐AN**, our observations highlight that both ActD and Flavopiridol induce distinct and specific morphological changes in nucleoli. In light of these findings, we anticipate that **Nu‐AN** will become a valuable tool for live cell nucleolar morphology, offering significant advantages for research in the field of nucleolar biology. We also hope that the design of such probes opens up new avenues for the development of probes targeting other membraneless organelles within cells. This will fill the visualization gap for membraneless organelles and enhance our overall understanding of cell biology.

## Conflict of Interest

The authors declare no conflict of interest.

## Supporting information

Supporting Information

## Data Availability

The data that support the findings of this study are available in the supplementary material of this article.
