# Supplementary material for: Rna Buffering Fluorogenic Probe for Nucleolar Morphology Stable Imaging And Nucleolar Stress‐Generating Agents Screening
Source: Adv Sci (Weinh). 2024 Feb 7;11(15):2309743. doi: 10.1002/advs.202309743 (PMC11022735; doi:10.1002/advs.202309743)
Supplement: Supplementary file 1 — Supporting Information [file ADVS-11-2309743-s001.pdf]

ADVANCED SCIENCE
Open Access

Supporting Information

for *Adv. Sci.*, DOI 10.1002/advs.202309743

Rna Buffering Fluorogenic Probe for Nucleolar Morphology Stable Imaging And Nucleolar Stress-Generating Agents Screening

Wenchao Jiang, Qinglong Qiao, Jie Chen, Pengjun Bao, Yi Tao, Yinchan Zhang and Zhaochao Xu**

RNA buffering fluorogenic probe for nucleolar morphology stable imaging and nucleolar stress-generating agents screening

Wenchao Jiang,[a, b] Qinglong Qiao,[a,] *Jie Chen, [a, b] Pengjun Bao,[a, b] Yi Tao,[a, b] Yinchan Zhang[a, b] and Zhaochao Xu[a, b,] *

[a] CAS Key Laboratory of Separation Science for Analytical Chemistry, Dalian Institute of Chemical Physics, Chinese Academy of Sciences, 457 Zhongshan Road, Dalian 116023 (China)
E-mail: qqlqiao@dicp.ac.cn; zcxu@dicp.ac.cn

[b] University of Chinese Academy of Sciences, Beijing 100049 (China)

Content

1. Computational Details and Molecular docking 5

1.1 Computational details 5

1.2 Molecular docking 5

2.Experimental details 5

2.1 Materials and instruments 5

2.2 Spectroscopic studies 6

2.3 Cell culture and transfection 7

3.Imaging 8

3.1 Confocal Imaging of living cell 8

3.2 Confocal Imaging of fixed cell 9

3.3 Confocal Imaging of Hela cell treated with anticancer drugs 9

3.4 Fluorescence recovery after photobleaching (FRAP) imaging 10

4 Image analysis and prediction of pK_a 10

3. Synthesis of Nu-AN 10

Scheme S1. The synthesis procedure of **Nu-AN** 11

5.Spectroscopic characterizations of Nu-AN 13

Figure S1. UV-Vis absorption spectra of 5 μM **Nu-AN** in different solvents(a) and a methanol-glycerol mixture with different volume fractions(b). 13

Table S1. Photophysical data for **Nu-AN** in different solvents: peak UV-vis absorption wavelength (λ_{abs}), maximum emission wavelength (λ_{em}), Stokes shifts(Δλ), molar absorption coefficient (ε) and fluorescence quantum yield (φ) 13

Table S2. The viscosity of glycerol/methanol binary mixture at various volume ratios of glycerol at 25°C,and the quantum yields of **Nu-AN** at different viscosities. 13

Table S3 The quantum yields (φ), molar absorption coefficient (ε) and brightness(φ*ε) of **Nu-AN** at different RNA concentrations 14

Figure S2. Corresponding quantum yields as a function of viscosity of **Nu-AN**. 14

6. Molecular docking 14

Figure S3. Five potential binding sites of 5ML7 defined by Schrödinger Maestro, the corresponding site scores. 15

7.Fluorescent imaging of live cells using Nu-AN 15

7.1. Colocalization imaging of Nu-AN in live cells 15

Figure S4.(a) Confocal images of Hela cells co-stained with 1 μM Lyso-Tracker Red and 2 μM Probe 1[3].Probe 1 displayed significant lysosomal fluorescence signal .(b) Confocal images of Hela cells co-stained with 1 μM Lyso-Tracker Red and 2 μM **Nu-AN**.(c) Confocal images of Hela cells co-stained with 1 μM Mito-Tracker Red and 2 μM **Nu-AN**. 15

7.2. Toxicity test of Nu-AN in Live Hela cells 16

Figure S5. Toxity test of **Nu-AN** with different concentrations in Hela cells 16

7.3. Fluorescent image of Hela cells incubated with Nu-AN in different concentrations. 16

Figure S6. Hela cells was incubated with **Nu-AN** in different concentrations for wash-free imaging.(Ex:488 nm, collected: 500-600 nm) 16

7.4. Fluorescent image of different cell lines incubated with Nu-AN 16

Figure S7. Hela, CoCa2, 293T, Vero cells was incubated with **Nu-AN** (2 μM)for wash-free imaging. (Ex:488 nm, collected: 500-600 nm) 16

7.5. Imaging of Nu-AN binding reversibly to the nucleolus 17

Figure S8. (a)Reversible stain of **Nu-AN** to the nucleolus.Imaging of living Hela cells stained with 3 μM **Nu-AN** ,or after washing out dyes,and 3 μM **Nu-AN** addition.(b) Average fluorescence intensity of a single nucleolus under three conditions. 17

7.6. FRAP imaging 17

Figure S9. Relative intensity is plotted versus time (s) in the region of interest during five cycles of FRAP processes and corresponding to the first and third images in each cycle process 17

8 Nucleolar Morphology Imaging and Screening of Drugs Inducing Nucleolar Stress 17

8.1.The selectivity of Nu-AN for nucleolar substructure 17

Figure S10. (a)(b)Overlay images of **Nu-AN** and NMP1-mCherry, corresponding normalized fluorescence intensity distribution in the region of interest.(c)(d) Overlay images of **Nu-AN** and FBL-mCherry, corresponding normalized fluorescence intensity distribution in the region of interest. The arrow indicates the difference in their intensity distribution. 18

8.2.Morphology of nucleoli in living Hela cells treated with anti-cancer drugs 18

Figure S11. (a)Morphology of nucleoli in living Hela cells treated with anti-cancer drugs and after drugs washout under confocal microscopy. NPM1-mCherry transiently expressing living Hela cells treated with 8 nM ActD for 4 h or 10 μM Flavopiridol for 1 h, and then co-stained with 2 μM **Nu-AN** for imaging. The drug was washed off and cultured Hela cell for another 4 hours, and then 2 μM **Nu-AN** was added for imaging.(b) Enlarged imagse of the region of interest in (a). 18

Figure S12. Morphology of nucleoli in living Hela cells treated with anti-cancer drugs and after drugs washout under confocal microscopy. FBL-mCherry transiently expressing living Hela cells treated with 8 nM ActD for 4 h or 10 μM Flavopiridol for 1 h, and then co-stained with 2 μM **Nu-AN** for imaging. The drug was washed off and cultured Hela cell for another 4 hours, and then 2 μM **Nu-AN** was added for imaging.. 19

8.3.Imaging of Hela cells treated with ActD for different times 19

Figure S13. Imaging of Hela cells treated with 8 nM ActD for different times and stained with 2 μM **Nu-AN** 20

Figure S14. (e) The ratio of the average fluorescence intensity of the nucleolus to the nucleoplasm with different treatment in living Hela cells. (f) The ratio of the average fluorescence intensity of the nucleolus to the extranucleolar cellular region with different treatment in living Hela cells. 20

8.4.Imaging Hela cells treated with Flavopiridol in different region 20

Figure S15. Confocal images of living Hela cells incubated with 10 μM Flavopiridol for 1 h and stained with 2 μM **Nu-AN**. Fluorescence field (left), overlapping fluorescence and bright fields(right). 21

9 ^{1}H-NMR, ^{13}C-NMR and HRMS spectra 21

Figure S16. ^{1}H-NMR spectrum of **pre-Nu-AN** in DMSO 21

Figure S17. ^{1}H-NMR spectrum of **Nu-AN** in DMSO 22

Figure S18. ^{13}C-NMR spectrum of **Nu-AN** in DMSO. 22

Figure S19. HRMS spectrum of **Nu-AN**. 23

10. References 24

1. Computational Details and Molecular docking

1.1 Computational details

All density functional theory (DFT) calculations were performed with *Gaussian 16* software.[1] The ground state geometries were optimized at the B3LYP/Def2-SVP level in water. Frequency calculation at the same level with the same basis set was performed to ensure that the geometries correspond to real minima on the potential energy surfaces (PES). *Multiwfn 3.8* was used to calculate MPP, SDP.[2]

1.2 Molecular docking

The geometries of **Nu-AN** has been optimized with *Gaussian 16* software. The optimized molecular structures was used as input for the molecular docking study to investigate their binding site within RNA using *Schrödinger Maestro 11.5 version*. The RNA as reported in the PDB structure, 5ML7 has been used in this study.[3] 5ML7 were prepared using protein preparation wizard[4]. Determination of highly potential binding sites of ligands on receptor was carried out using SiteMap tool analysis[5]. Select the binding site with the highest score, then using Glide application, receptor grid was generated (assignment of ligand binding site for docking). All parameters were set to defaults for the Glide docking process. 2D diagram of interactions between **Nu-AN** and RNA was generated by software.[6]

2.Experimental details

2.1 Materials and instruments

All common reagents were purchased from commercial suppliers (Sigma-Aldrich, J&K, Innochem, and Aladdin) and used without further purification. Solvents such as

dimethyl sulfoxide (DMSO), methanol, ethanol, chloroform, and acetonitrile were purchased from J&K; silica gel (200-300 mesh) was purchased from Innochem. RNase (DNase free) , Dnase (Rnase free) and Deoxyribonucleic acid−cellulose double-stranded (dsDNA) from calf thymus DNA, were purchased from Sigma-Aldrich. single-stranded DNA from salmon sperm was purchased from meilunbio(Dalian, China). Yeast ribonucleic acid (RNA) was purchased from absin (Shanghai, China).The plasmid encoding the NPM1-mCherry and FBL-mCherry fusion protein was provided by Prof. Yang Wang, Dalian Medical University.

The ^{1}H-NMR and ^{13}C-NMR spectra were recorded on a Bruker 400 spectrometer, with tetramethylsilane (TMS) as the internal standard. Chemical shifts were given in ppm and coupling constants (J) in Hz. High-resolution mass spectrometry (HRMS) data were recorded using ESI (6540 UHD Q-TOF, positive ion). UV-vis absorption spectra were collected on an Agilent Cary 60 UV-Vis Spectrophotometer. Fluorescence measurements were observed on an Agilent CARY Eclipse fluorescence spectrophotometer. Confocal images were performed on Olympus FV1000 MPE with a microscope IX 71, a 100× / NA 1.40 oil objective lens, LU-NV series laser unit (laser combination: 405nm; 488 nm; 543 nm). The single-photon confocal microscope system was built on an Olympus inverted microscope IX81.

2.2 Spectroscopic studies

2.2.1 Spectral measurements in the different solvents

The stock solution (2 mM) of **Nu-AN** was prepared in DMSO. The UV−vis absorption and fluorescence spectra of **Nu-AN** were measured in different solvents, including toluene, $CHCl_3$, DCM, Dioxane, EA, EtOH, MeCN, MeOH, DMSO, H_2O,CD_3OD, and D_2O at a concentration of 5 μM.

The viscosity response of the dye was carried out at 25 °C. In this experiment, the concentration of the dye solution was 5 μM, and the dyes were dissolved in a methanol-glycerol mixture with different volume fractions.

Coumarin 153 (0.53 in ethanol) were used to obtain fluorescence quantum yields of other compounds using the relative determination method. The quantum yield (φ) was calculated using the following equation[7]:

$$\varphi F_{(X)} = \varphi F_{(S)} \cdot (A_S F_X / A_X F_S)(\eta_X / \eta_S)^2$$

where φF, A, and F represent the fluorescence quantum yield, the absorbance at the excitation wavelength, and the area under the corrected emission curve. η is the refractive index of the solvent. Subscripts X and S refer to the unknown and the standard samples, respectively.

2.2.2 Spectral measurements in the presence of nucleic acids

Yeast RNA, ssDNA, and dsDNA are respectively dissolved in TE buffer (Tris-EDTA sterile solution pH = 7.4) to prepare nucleic acid stock solution. Test nucleic acid concentration by spectrophotometer: RNA (42.2 mg/mL), ssDNA (27 mg/mL), dsDNA (2.5 mg/mL). RNA stock solution was dissolved in Phosphate buffer (10 mM, pH = 7.4) to prepare RNA solutions with different concentrations, to which **Nu-AN** was added with concentration of 5 μM, and the UV−vis absorption and fluorescence spectra were recorded. Fluorescence titration of **Nu-AN** (5 μM) with ssDNA and dsDNA were performed according to the testing method of RNA.

2.3 Cell culture and transfection

2.3.1 Cell culture

Hela (helacyton gartleri), CoCa2,HEK293T, Vero cells, were purchased from the Cell Bank of Type Culture Collection of the Chinese Academy of Sciences. Cells were maintained in Dulbecco's modified Eagle's medium (DMEM, Gibco) supplemented with 10% fetal bovine serum (FBS, Hyclone) which were cultured in a humidified atmosphere of 5% CO_2/95% air at 37 °C. Before the imaging experiments, Hela cells were seeded on a glass-bottom cell culture dish (Nest, polystyrene, Φ 15 mm) for 1-2 days to reach 40-90% confluency. The cells were then used for further experiments.

2.3.2 Cell transfection

Transfection experiment was performed according Lipofectamine 2000 (Invitrogen) according to the manufacturer's protocol.

Briefly, 1.5 μL Lipofectamine 2000 (Invitrogen) and appropriate plasmid were firstly diluted in 20 μL DMEM (dulbecco's modified eagle medium), respectively. 5 min later, the diluted plasmid in 20 μL DMEM was added to the diluted Lipofectamine 2000 (Invitrogen) with homogeneous mixing. Another 10 min later, the mixture was added to the cell-culture dish in 1 mL DMEM. The final concentration of plasmid was controlled at 500-1000 ng/mL. After incubated 4 h in 37 °C, the culture medium was changed from DMEM to DMEM with 10% FBS. 24 - 48 h later, the transfected cells were used for imaging.

3.Imaging

3.1 Confocal Imaging of living cell

Generally, **Nu-AN** and organelles tracker were pre-incubated with live Hela cells for 30 min in a humidified atmosphere of 5% CO_2/95% air at 37 °C. Then, the co-

localization imaging was performed using Olympus FV1000 at room temperature. The pixel size of the images is 1024*1024. The nucleus was stained with Hoechst 33342 (3 μM). Mitochondria were stained with Mito-Tracker Red (0.5 μM). Lysosome were stained with Lyso-Tracker Red (1 μM). Ex:405 nm, collected: 425-475 nm for Hoechst 33342; Ex:488 nm, collected: 495-535 nm for **Nu-AN**; Ex:543nm, collected: 550-650 nm for Mito Tracker Red and Lyso-Tracker Red. Staining of the GC by **Nu-AN** was demonstrated by colocalization with NPM1 or FBL labeled with mCherry. Ex:488 nm, collected: 495-535 nm for **Nu-AN**; Ex:543nm, collected: 550-650 nm for mCherry.

3.2 Confocal Imaging of fixed cell

The growth medium was removed and the cells were fixed in pre-chilled methanol at - 20 ^{o}C for 10 min. After the cells were washed in PBS buffer (1 mL) for three times, the cells were incubated with dyes for 0.5 h (**Nu-AN**: 2 μM, Hoechst 33342: 3 μM) before imaging.

Fixed Hela Cells treated with Ribonuclease (RNase) or Deoxyribonuclease (DNase): after fixed Hela cells, a solution of RNase (100 ug mL^{-1}) or DNase (100 U mL^{-1}) in PBS buffer was applied and the cells were incubated at 37 ^{o}C for 2 h. After RNase or DNase digestion, the enzyme solution was removed and the cells were washed in PBS buffer (1 mL) for three times, the incubated with 2 μM **Nu-AN** for imaging.

3.3 Confocal Imaging of Hela cell treated with anticancer drugs

Hela cell treated with Actinomycin D (8 nM) or Flavopiridol (10 μM) and the cells were incubated at 37 ^{o}C for different times. After incubation, the cell were directly incubated with dyes for wash-free imaging, or the cell were fixed and incubated with dyes for wash-free imaging.

3.4 Fluorescence recovery after photobleaching (FRAP) imaging

Fluorescence recovery after photobleaching (FRAP) experiments were carried out on Olympus FV1000 MPE confocal laser scanning microscope. Two images were taken before the bleach pulse and 25 images after the bleaching of regions of interest (ROIs) at 6 μW laser transmission to minimize scan bleaching. For photobleaching process, the ROIs were bleached by laser for 10 seconds. The fluorescence intensity was acquired by the software in imaging system.

4 Image analysis and prediction of pK_a

Image processing and fluorescence intensity analysis were conducted by ImageJ.

Marvin was used for drawing and predicting pK_a of **Nu-AN**, Marvin 17.21.0, Chemaxon (https://www.chemaxon.com). Epik (Schrödinger, http://www.schrodinger.com) was also used for predicting pK_a of **Nu-AN**.

3. Synthesis of Nu-AN

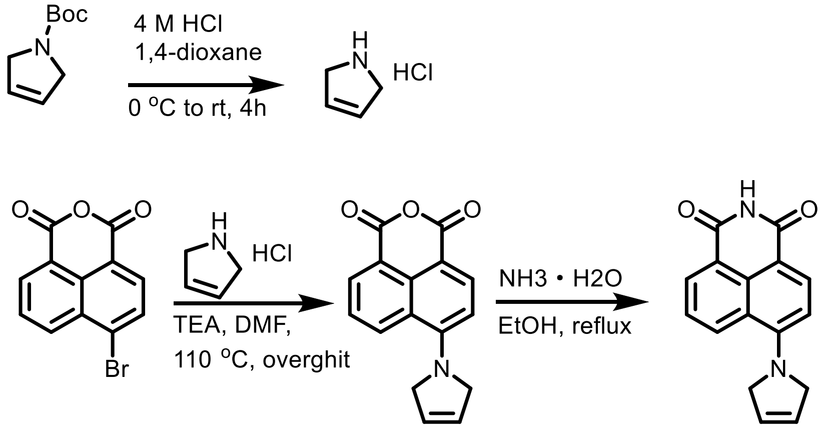

pre-Nu-AN **Nu-AN**

Scheme S1. The synthesis procedure of ***Nu-AN***

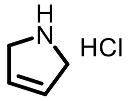

3-Pyrroline hydrochloride: N-Boc-3-Pyrroline (10 g) was dissolved in dioxane, dropping hydrogen chloride (20 eq, 4 M in dioxane) in an ice bath. After the addition was complete, the reaction was stirred at room temperature for 4 h. The reaction solution was suction filtered to collect the solid, and further vacuum-dried to obtain 3-Pyrroline hydrochloride, a brown solid (5.8 g, yield 93.3%).The crude product was used in the next step without further purification or characterization.

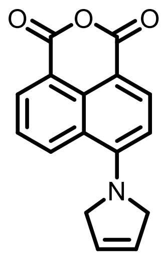

6-(2,5-dihydro-1H-pyrrol-1-yl)-1H,3H-benzo[de]isochromene-1,3-dione(pre-Nu-AN): A solution of 4-Bromo-1,8-naphthalic anhydride (1 g, 3.62 mmol, 1 eq.), 3-Pyrroline hydrochloride (1.9 g, 18.1 mmol, 5 eq.), Triethylamine (3 mL, 21,7 mmol, 6 eq.) in DMF (3 mL) was heated to 110°C and stirred at this temperature overghit in a sealed tube.The mixture was cooled to room temperature, removing the solvent

under reduced pressure and the residue was further purified by flash column chromatography (DCM/MeOH = 20/1) to obtain a yellow solid (550 mg,yield 57.2%).

^{1}H NMR (400 MHz, DMSO) δ 9.03 (d, J = 8.6 Hz, 1H), 8.48 (d, J = 7.4 Hz, 1H), 8.29 (d, J = 8.9 Hz, 1H), 7.77 – 7.62 (m, 1H), 6.89 (d, J = 8.9 Hz, 1H), 6.15 (s, 2H), 4.75 (s, 4H).

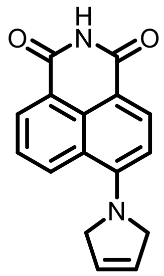

6-(2,5-dihydro-1H-pyrrol-1-yl)-1H-benzo[de]isoquinoline-1,3(2H)-dione (Nu-AN): pre-3-Pyr-Nap (30 mg,0.113 mmol, 1 eq) was dissolved in ethanol, to which was added excess ammonia (~30 eq) and stirred at reflux until the starting material was consumed and monitored by TLC. The mixture was cooled to room temperature, removing the solvent under reduced pressure and the residue was further purified by flash column chromatography (DCM/MeOH = 30/1) to obtain an orange solid (21 mg,yield 70.3%)

^{1}H NMR (400 MHz, DMSO) δ 11.35 (s, 1H), 8.92 (d, J = 8.7 Hz, 1H), 8.42 (d, J = 7.2 Hz, 1H), 8.24 (d, J = 8.7 Hz, 1H), 7.64 (s, 1H), 6.85 (d, J = 8.8 Hz, 1H), 6.13 (s, 2H), 4.69 (s, 4H).^{13}C NMR (101 MHz, DMSO) δ 165.02, 164.06, 151.12, 133.06, 132.77, 132.48, 130.37, 126.00, 123.83, 122.69, 121.88, 109.63, 109.09, 59.58. HRMS (ESI) Exact mass calculated for $C_{16}H_{13}N_2O_2$ $[M+H]^+$: 265.0972, found: 265.0972.

5.Spectroscopic characterizations of Nu-AN

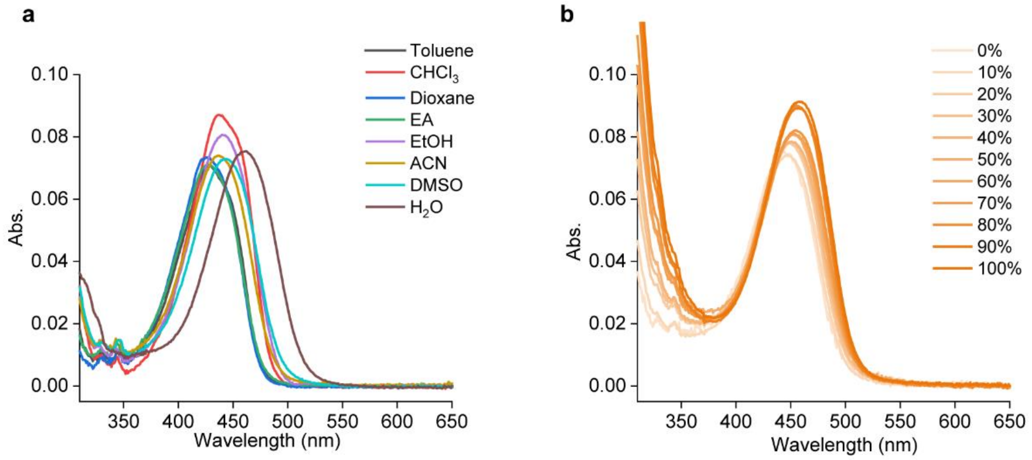

Figure S1. UV-Vis absorption spectra of 5 μM ***Nu-AN*** *in different solvents(a) and a methanol-glycerol mixture with different volume fractions(b).*

Table S1. Photophysical data for ***Nu-AN*** *in different solvents: peak UV-vis absorption wavelength (λ_{abs}), maximum emission wavelength (λ_{em}), Stokes shifts($\Delta\lambda$), molar absorption coefficient (ε) and fluorescence quantum yield (φ)*

solvent	λ_{abs}	λ_{em}	$\Delta\lambda$	ε	φ
Toluene	430	485	55	14124	0.70
$CHCl_3$	437	496	59	17416	0.79
Dioxane	426	492	66	14686	0.65
EA	427	498	71	14218	0.25
ACN	437	512	75	14792	0.042
DMSO	443	511	68	14582	0.031
EtOH	440	514	74	16134	0.025
MeOH	444	536	92	16288	0.014
H_2O	462	552	90	15090	0.007
CD_3OD	443	534	91	15810	0.020
D_2O	461	541	80	15122	0.015

Table S2. The viscosity of glycerol/methanol binary mixture at various volume ratios of glycerol at 25°C,and the quantum yields of ***Nu-AN*** *at different viscosities.*

Glycerol %	0	10	20	30	40	50	60	70	80	90	100
Viscosity (cP)	0.53	0.92	1.58	2.97	5.6	11.18	24.52	53.27	135.72	366.45	920.88
φ	0.021	0.022	0.020	0.023	0.026	0.027	0.035	0.046	0.054	0.087	0.152

*Table S3 The quantum yields (φ), molar absorption coefficient (ε) and brightness(φ*ε) of **Nu-AN** at different RNA concentrations*

RNA concentration (ug/mL)	φ	ε (M^{-1}·cm^{-1}))	brightness (φ*ε)
0	0.006	16272	98.6
53.25	0.019	15128	282.9
106.5	0.030	14660	435.6
213	0.053	14492	761.4
426	0.092	13356	1234.1
639	0.107	13754	1466.7
852	0.131	12850	1683.9
1065	0.142	12588	1786.9
1278	0.161	12010	1932.4
1491	0.160	12562	2005.0
1704	0.171	12552	2141.8
1917	0.167	12406	2067.0
2130	0.183	11774	2158.4

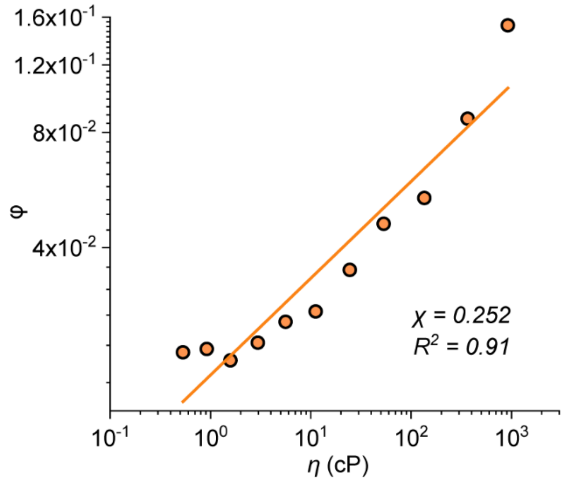

*Figure S2. Corresponding quantum yields as a function of viscosity of **Nu-AN**.*

6. Molecular docking

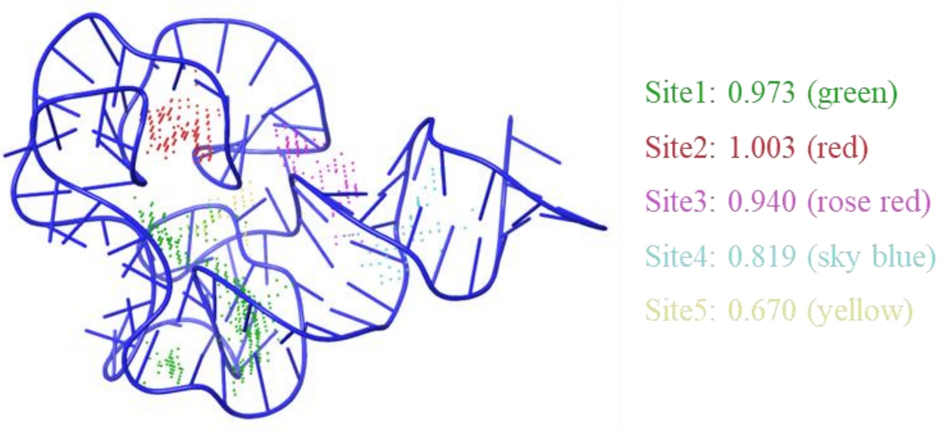

Figure S3. Five potential binding sites of 5ML7 defined by Schrödinger Maestro, the corresponding site scores.

7.Fluorescent imaging of live cells using Nu-AN

7.1. Colocalization imaging of Nu-AN in live cells

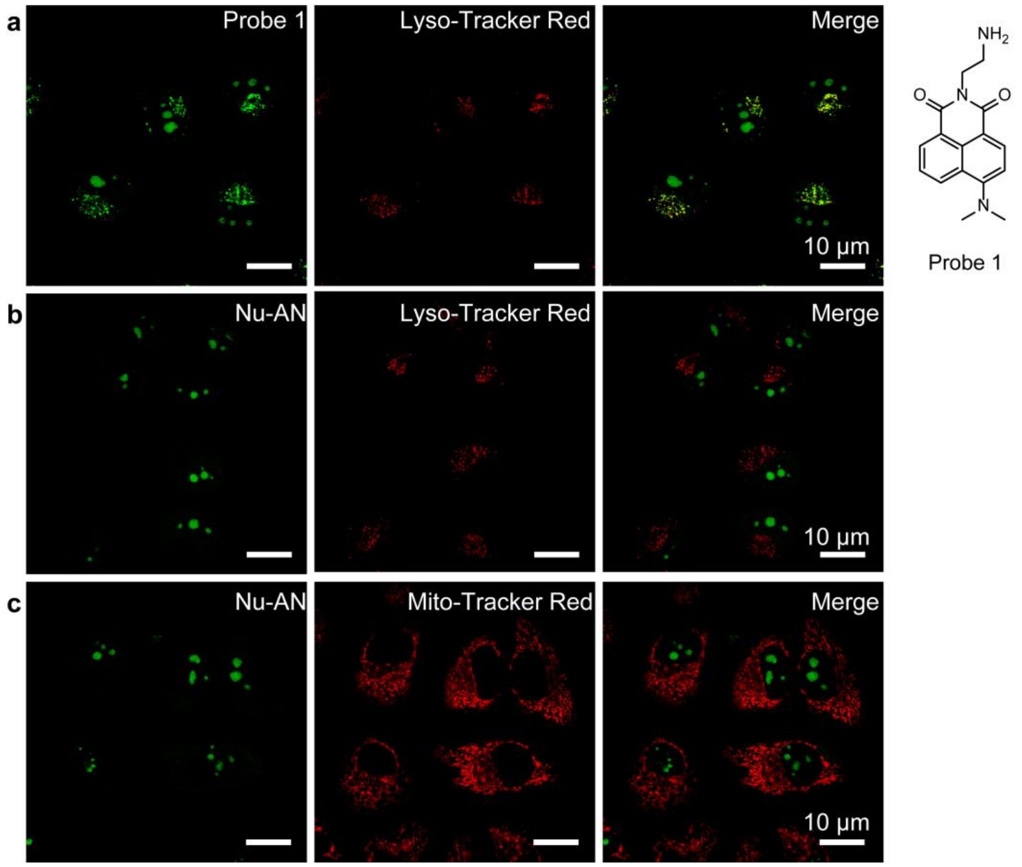

Figure S4.(a) Confocal images of Hela cells co-stained with 1 μM Lyso-Tracker Red and 2 μM Probe 1[3].Probe 1 displayed significant lysosomal fluorescence signal .(b) Confocal images of Hela cells co-stained with 1 μM Lyso-Tracker Red and 2 μM ***Nu-AN****.(c) Confocal images of Hela cells co-stained with 1 μM Mito-Tracker Red and 2 μM* ***Nu-AN****.*

7.2. Toxicity test of Nu-AN in Live Hela cells

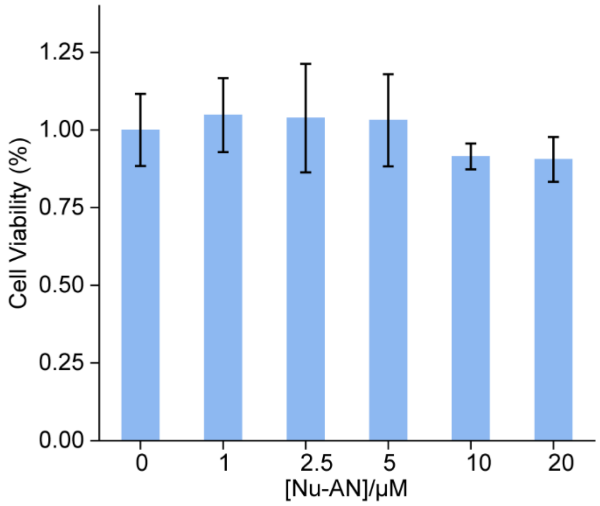

*Figure S5. Toxity test of **Nu-AN** with different concentrations in Hela cells*

7.3. Fluorescent image of Hela cells incubated with Nu-AN in different concentrations.

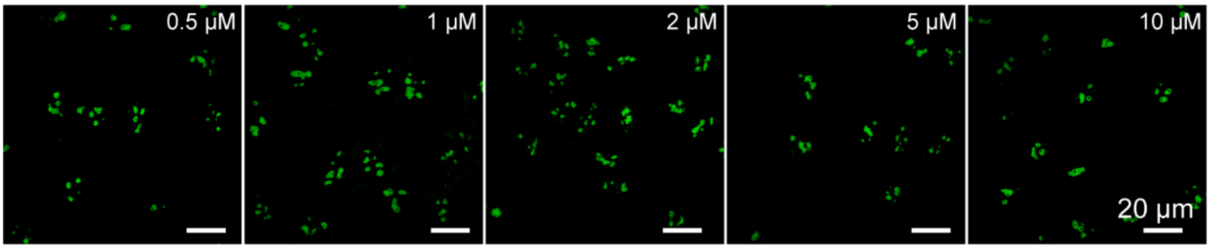

*Figure S6. Hela cells was incubated with **Nu-AN** in different concentrations for wash-free imaging.(Ex:488 nm, collected: 500-600 nm)*

7.4. Fluorescent image of different cell lines incubated with Nu-AN

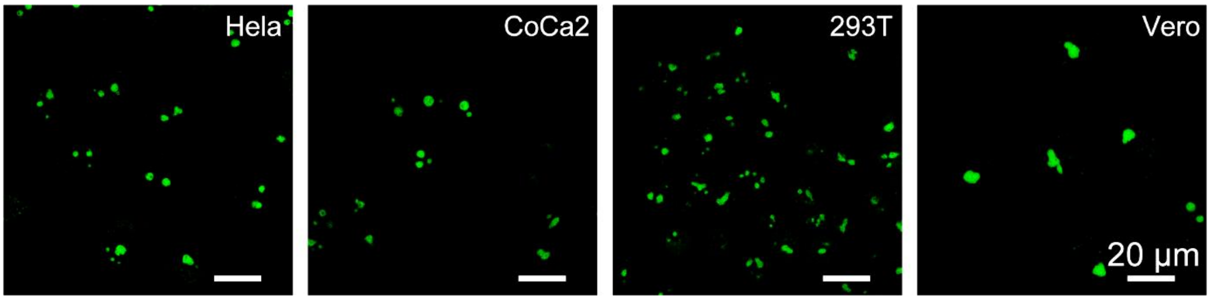

*Figure S7. Hela, CoCa2, 293T, Vero cells was incubated with **Nu-AN** (2 μM)for wash-free imaging. (Ex:488 nm, collected: 500-600 nm)*

7.5. Imaging of Nu-AN binding reversibly to the nucleolus

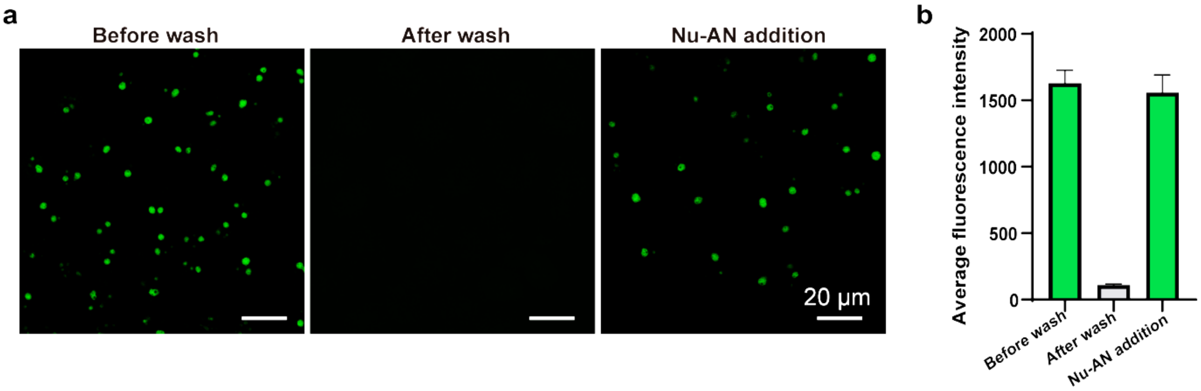

*Figure S8. (a)Reversible stain of **Nu-AN** to the nucleolus.Imaging of living Hela cells stained with 3 μM **Nu-AN** ,or after washing out dyes,and 3 μM **Nu-AN** addition.(b) Average fluorescence intensity of a single nucleolus under three conditions.*

7.6. FRAP imaging

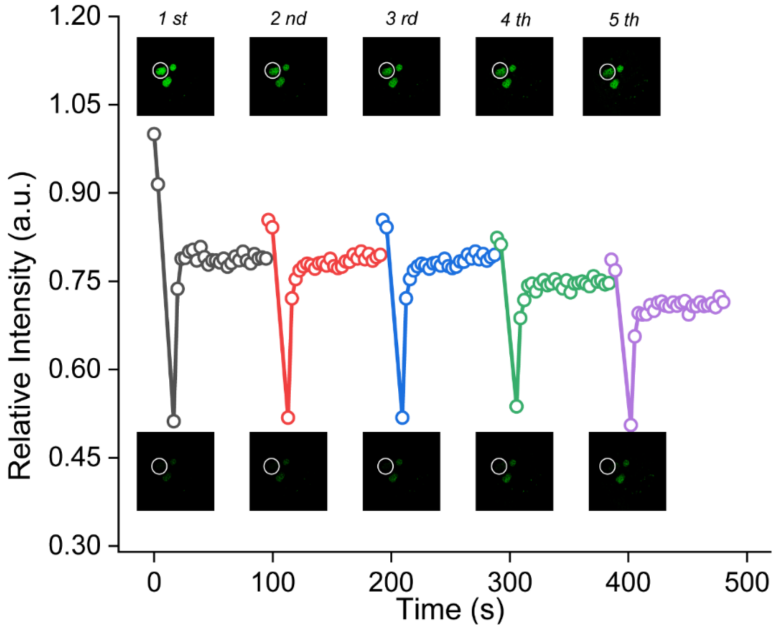

Figure S9. Relative intensity is plotted versus time (s) in the region of interest during five cycles of FRAP processes and corresponding to the first and third images in each cycle process

8 Nucleolar Morphology Imaging and Screening of Drugs Inducing Nucleolar Stress

8.1.The selectivity of Nu-AN for nucleolar substructure

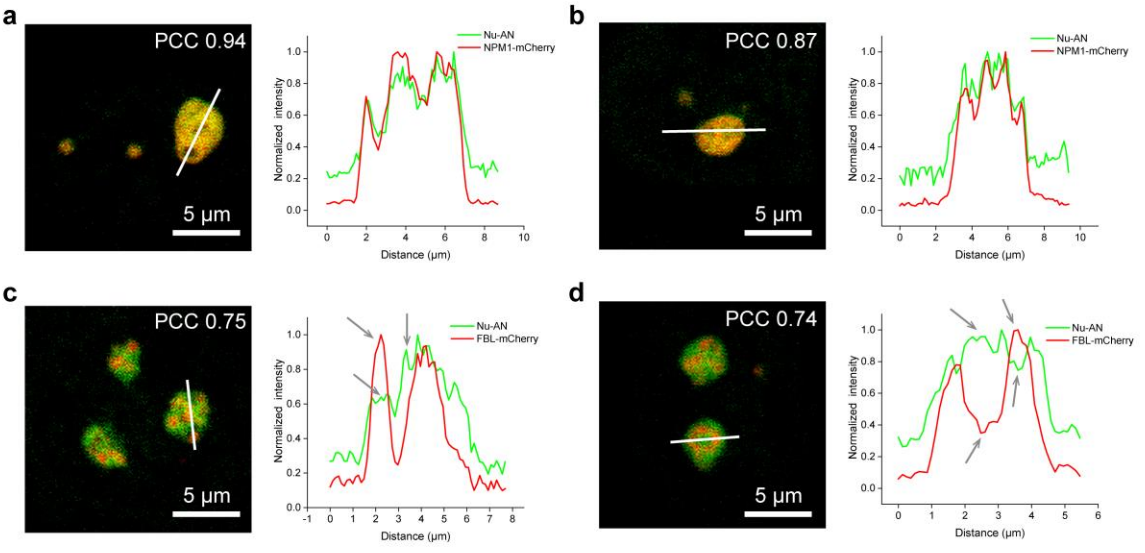

Figure S10. (a)(b)Overlay images of ***Nu-AN*** *and NMP1-mCherry, corresponding normalized fluorescence intensity distribution in the region of interest.(c)(d) Overlay images of* ***Nu-AN*** *and FBL-mCherry, corresponding normalized fluorescence intensity distribution in the region of interest. The arrow indicates the difference in their intensity distribution.*

8.2.Morphology of nucleoli in living Hela cells treated with anti-cancer drugs

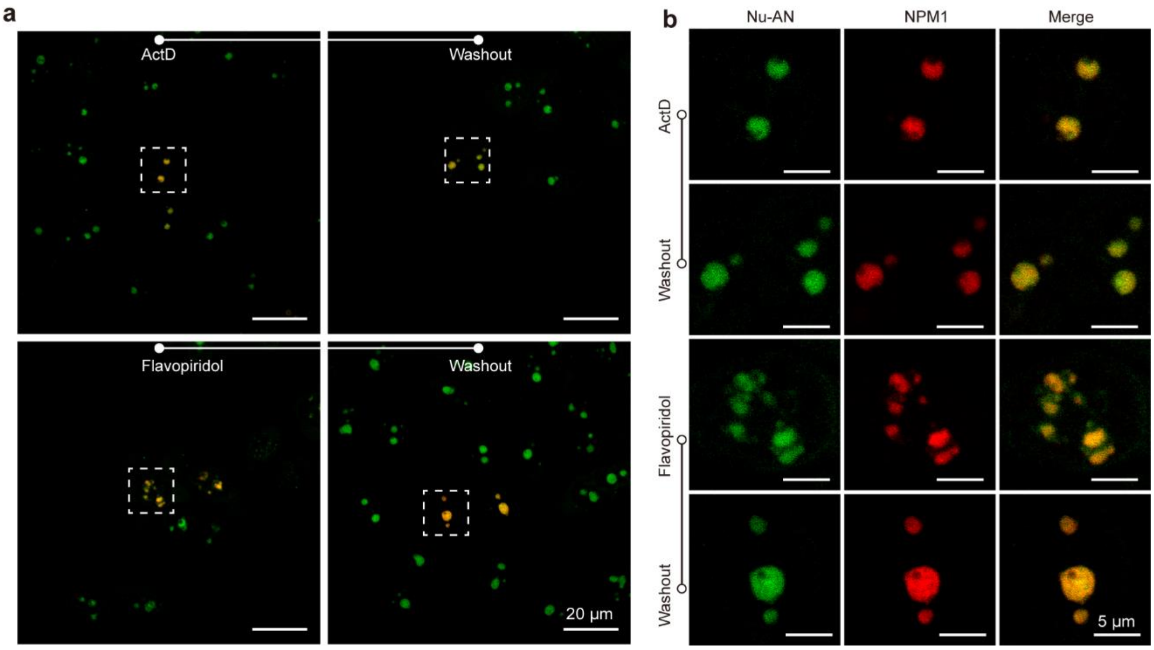

Figure S11. (a)Morphology of nucleoli in living Hela cells treated with anti-cancer drugs and after drugs washout under confocal microscopy. NPM1-mCherry transiently expressing living Hela cells treated with 8 nM ActD for 4 h or 10 μM Flavopiridol for 1 h, and then co-stained with 2 μM ***Nu-AN*** *for imaging. The drug was washed off and cultured Hela cell for another 4 hours, and then 2 μM* ***Nu-AN*** *was added for imaging.(b) Enlarged imagse of the region of interest in (a).*

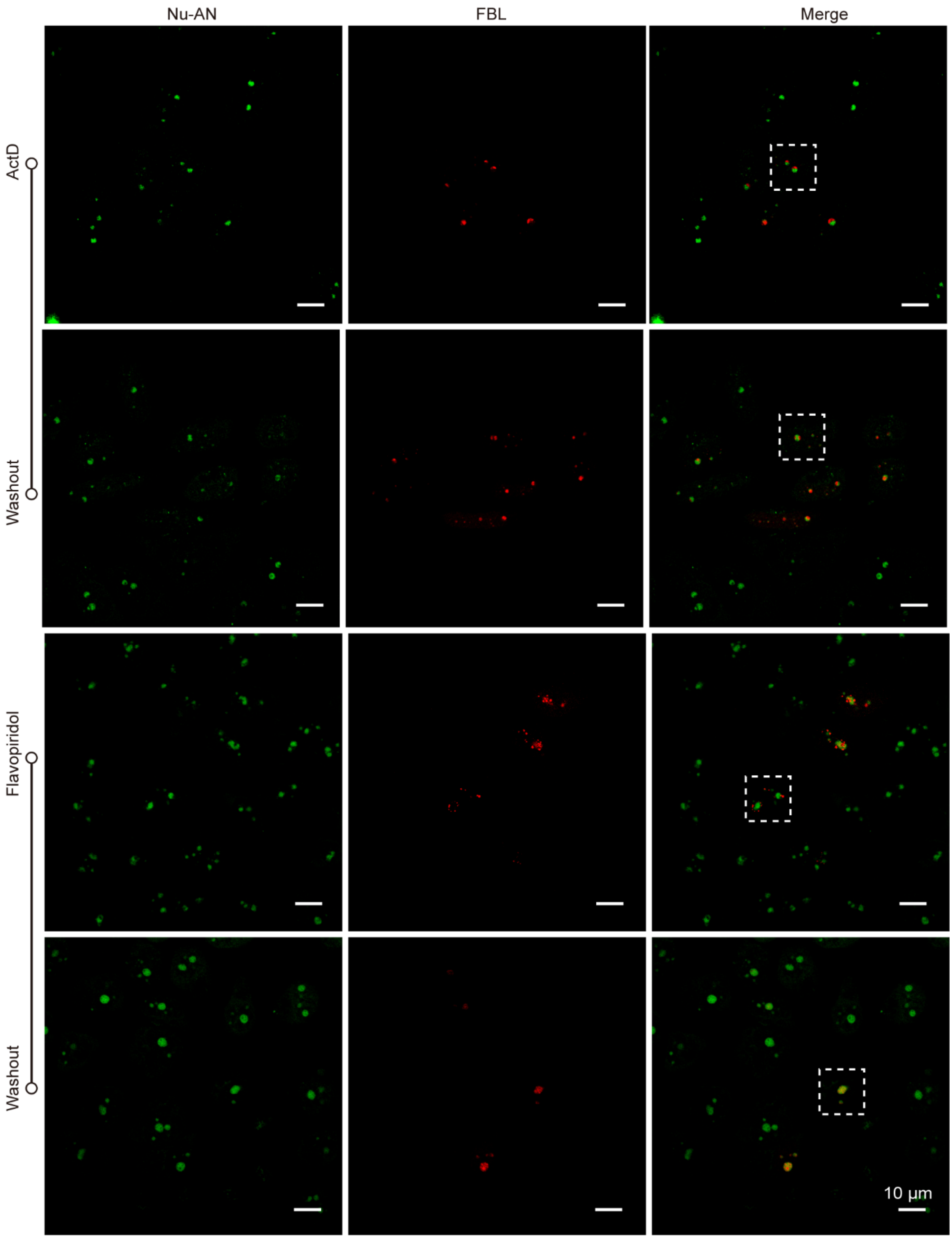

*Figure S12. Morphology of nucleoli in living Hela cells treated with anti-cancer drugs and after drugs washout under confocal microscopy. FBL-mCherry transiently expressing living Hela cells treated with 8 nM ActD for 4 h or 10 μM Flavopiridol for 1 h, and then co-stained with 2 μM **Nu-AN** for imaging. The drug was washed off and cultured Hela cell for another 4 hours, and then 2 μM **Nu-AN** was added for imaging*

8.3.Imaging of Hela cells treated with ActD for different times

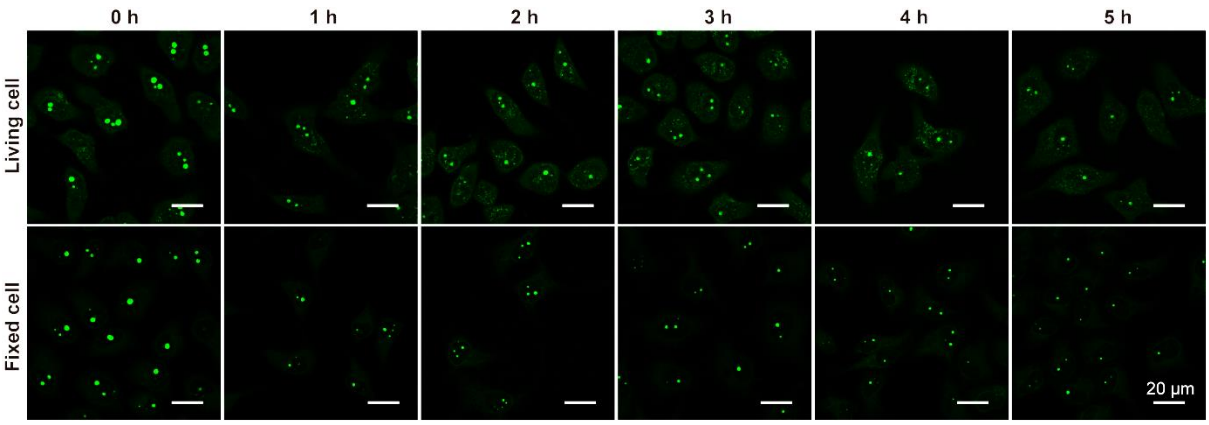

Figure S13. Imaging of Hela cells treated with 8 nM ActD for different times and stained with 2 μM ***Nu-AN***

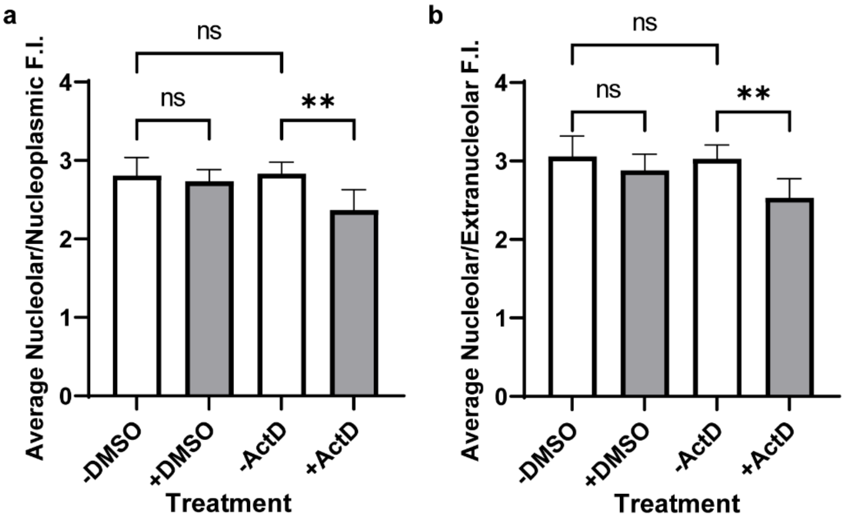

Figure S14. (e) The ratio of the average fluorescence intensity of the nucleolus to the nucleoplasm with different treatment in living Hela cells. (f) The ratio of the average fluorescence intensity of the nucleolus to the extranucleolar cellular region with different treatment in living Hela cells.

8.4.Imaging Hela cells treated with Flavopiridol in different region

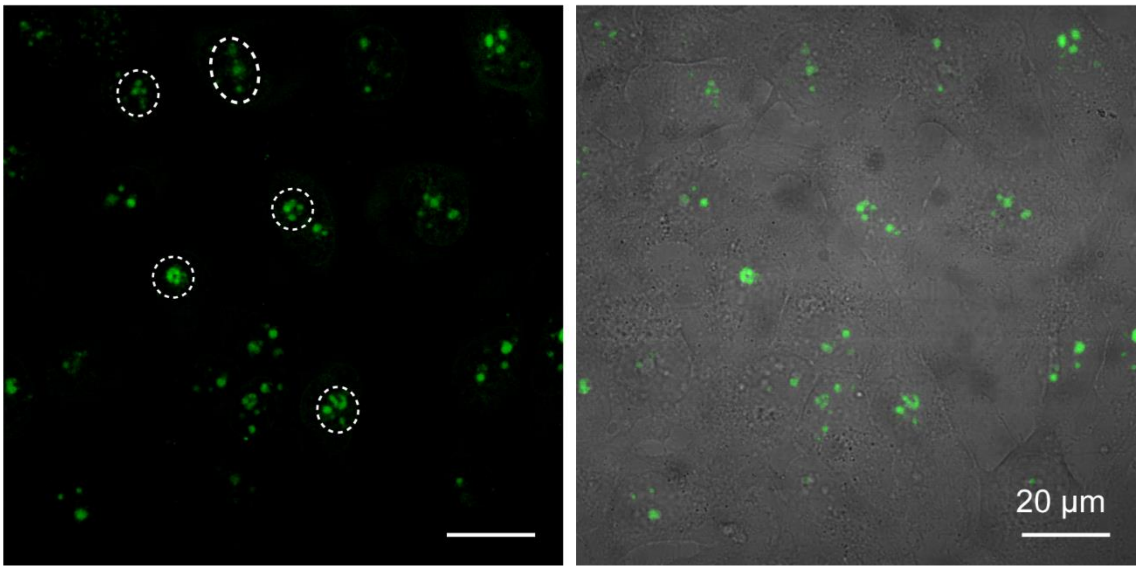

Figure S15. Confocal images of living Hela cells incubated with 10 μM Flavopiridol for 1 h and stained with 2 μM ***Nu-AN****. Fluorescence field (left), overlapping fluorescence and bright fields(right).*

9 ^{1}H-NMR, ^{13}C-NMR and HRMS spectra

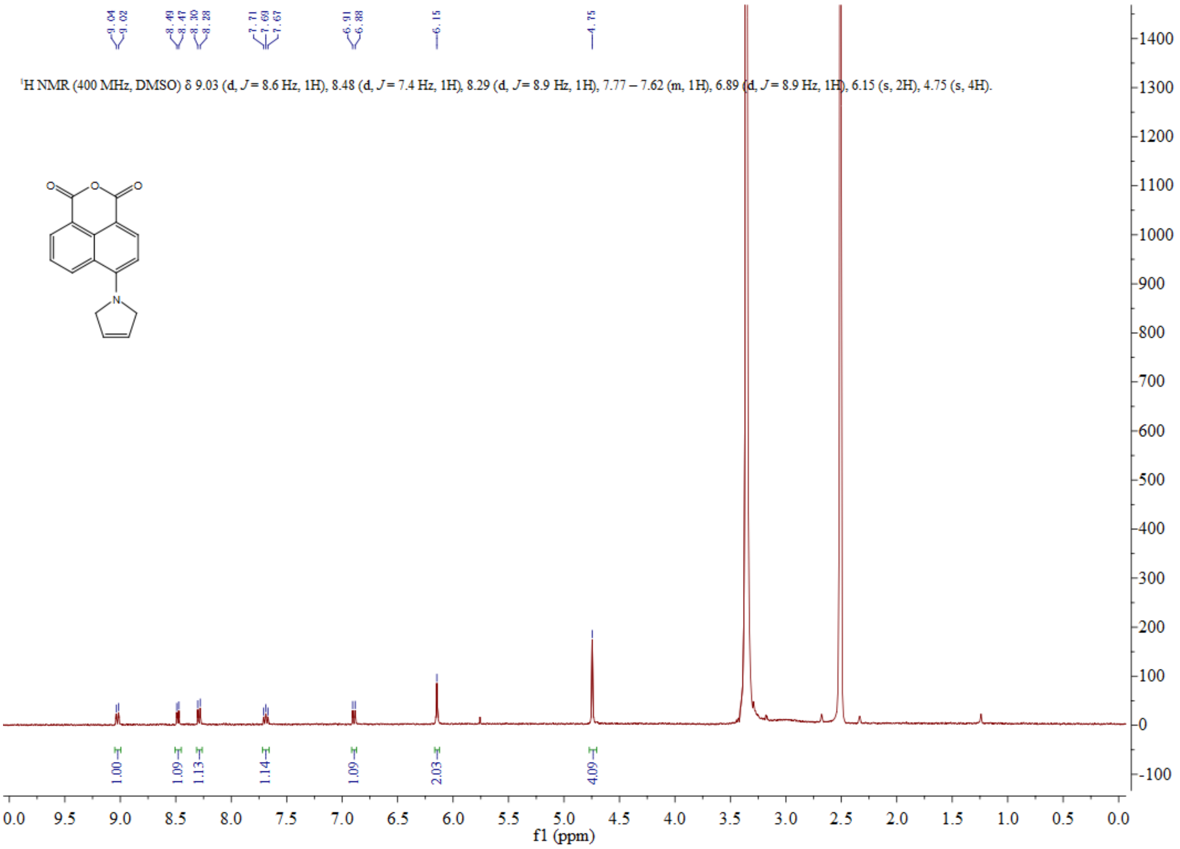

Figure S16. ^{1}H-NMR spectrum of ***pre-Nu-AN*** *in DMSO*

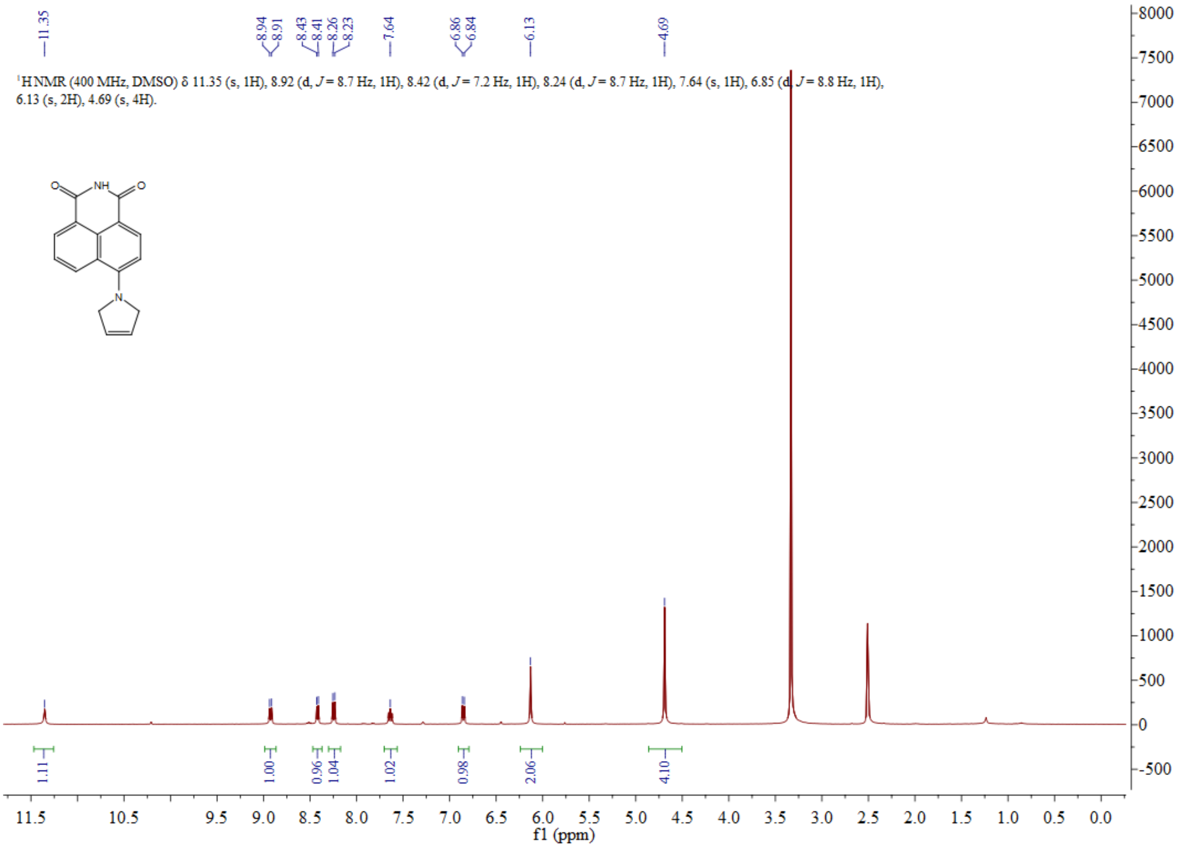

Figure S17. ^{1}H-NMR spectrum of **Nu-AN** in DMSO.

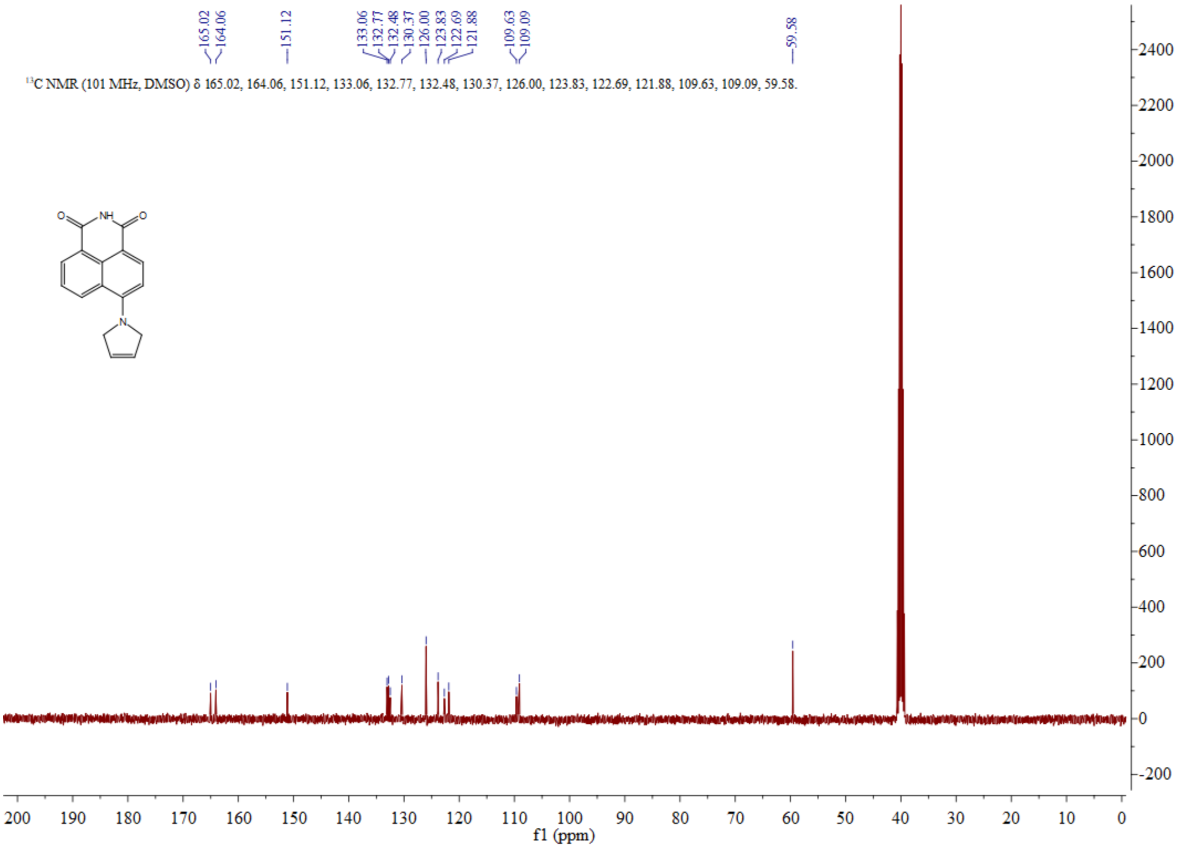

Figure S18. ^{13}C-NMR spectrum of **Nu-AN** in DMSO.

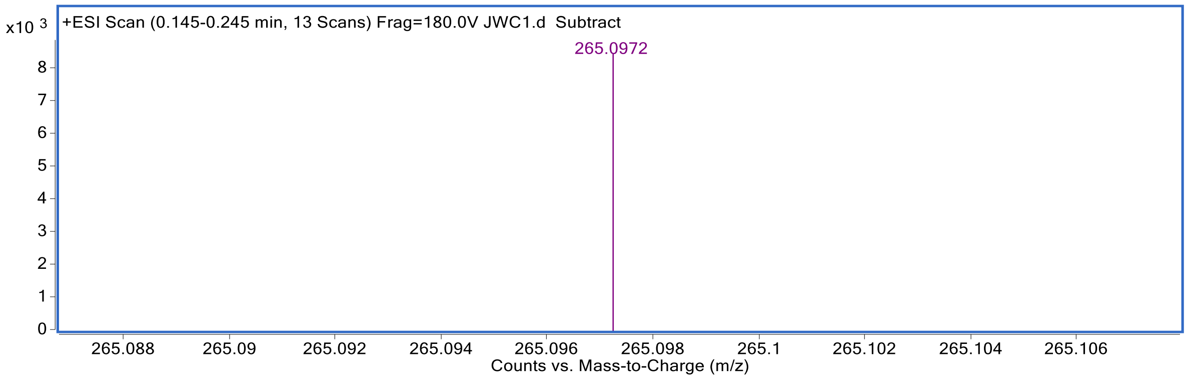

*Figure S19. HRMS spectrum of **Nu-AN**.*

10. References

(1) Frisch, M. J.; Trucks, G. W.; Schlegel, H. B.; Scuseria, G. E.; Robb, M. A.; Cheeseman, J. R.; Scalmani, G.; Barone, V.; Petersson, G. A.; Nakatsuji, H.; Li, X.; Caricato, M.; Marenich, A. V.; Bloino, J.; Janesko, B. G.; Gomperts, R.; Mennucci, B.; Hratchian, H. P.; Ortiz, J. V.; Izmaylov, A. F.; Sonnenberg, J. L.; Williams; Ding, F.; Lipparini, F.; Egidi, F.; Goings, J.; Peng, B.; Petrone, A.; Henderson, T.; Ranasinghe, D.; Zakrzewski, V. G.; Gao, J.; Rega, N.; Zheng, G.; Liang, W.; Hada, M.; Ehara, M.; Toyota, K.; Fukuda, R.; Hasegawa, J.; Ishida, M.; Nakajima, T.; Honda, Y.; Kitao, O.; Nakai, H.; Vreven, T.; Throssell, K.; Montgomery Jr., J. A.; Peralta, J. E.; Ogliaro, F.; Bearpark, M. J.; Heyd, J. J.; Brothers, E. N.; Kudin, K. N.; Staroverov, V. N.; Keith, T. A.; Kobayashi, R.; Normand, J.; Raghavachari, K.; Rendell, A. P.; Burant, J. C.; Iyengar, S. S.; Tomasi, J.; Cossi, M.; Millam, J. M.; Klene, M.; Adamo, C.; Cammi, R.; Ochterski, J. W.; Martin, R. L.; Morokuma, K.; Farkas, O.; Foresman, J. B.; Fox, D. J. *Gaussian 16 Rev. C.01*, Wallingford, CT, 2016.

(2) Lu, T. Simple, reliable, and universal metrics of molecular planarity. *J. Mol. Model.* **2021**, *27*, 263-268.

(3) Cao, C.; Wei, P.; Li, R.; Zhong, Y.; Li, X.; Xue, F.; Shi, Y.; Yi, T. Ribosomal RNA-Selective Light-Up Fluorescent Probe for Rapidly Imaging the Nucleolus in Live Cells. *ACS Sens.* **2019**, *4*, 1409-1416.

(4) Sastry, G. M.; Adzhigirey, M.; Day, T.; Annabhimoju, R.; Sherman, W. Protein and ligand preparation: parameters, protocols, and influence on virtual screening enrichments. *J. Comput. Aided Mol. Des.* **2013**, *27*, 221-34.

(5) Halgren, T. A. Identifying and Characterizing Binding Sites and Assessing Druggability. *J. Chem. Inf. Model.* **2009**, *49*, 377-389.

(6) Pattar, S. V.; Adhoni, S. A.; Kamanavalli, C. M.; Kumbar, S. S. In silico molecular docking studies and MM/GBSA analysis of coumarin-carbonodithioate hybrid derivatives divulge the anticancer potential against breast cancer. *Beni-Suef University Journal of Basic and Applied Sciences* **2020**, *9*.

(7) Wurth, C.; Grabolle, M.; Pauli, J.; Spieles, M.; Resch-Genger, U. Relative and absolute determination of fluorescence quantum yields of transparent samples. *Nat Protoc* **2013**, *8*, 1535-50.
